# Supplementary material for: Novel allogeneic CAR T-cell platform involving microhomology-mediated end joining repair and low off-targeting potential
Source: Mol Ther Nucleic Acids. 2025 Nov 17;36(4):102778. doi: 10.1016/j.omtn.2025.102778 (PMC12704299; doi:10.1016/j.omtn.2025.102778)
Supplement: Document S1. Figures S1–S6 and Table S1 [file mmc1.pdf]

**OMTN, Volume *36***

## Supplemental information

# Novel allogeneic CAR T-cell platform involving microhomology-mediated end joining repair and low off-targeting potential

**Tanya Hundal, Yan Luo, Yaqing Qie, Martha E. Gadd, Andrew D. Brim, Isas Vazquez-Rosario, Shaohua Guo, Mohamed A. Kharfan-Dabaja, and Hong Qin**

## Supplemental figures and legends

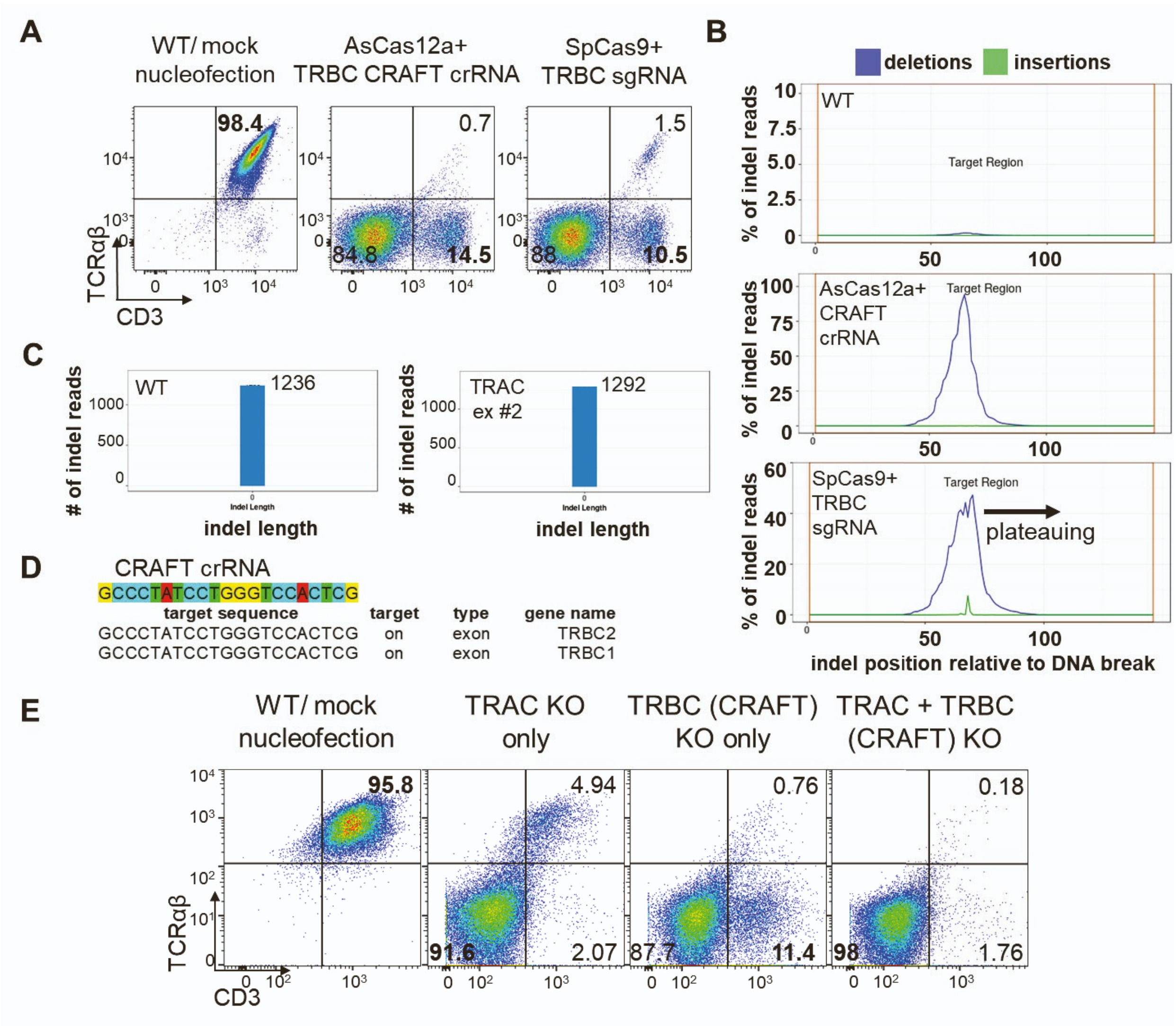


**Figure S1. AsCas12a/CRAFT crRNA-assisted TCR β editing has an improved indel profile over comparable SpCas9 editing, *related to Figure 1*. A** Protein expression showing generation of CRAFT-cells after TCR β KO via two strategies: AsCas12a Ultra/ TRBC crRNA (CRAFT crRNA) pair or via SpCas9/ TRBC sgRNA pair. Both pairs efficiently abrogated TCR and yielded similar percentages of TCR-,CD3+ CRAFT-cells, as determined by flow cytometry. **B** Graphs show the relative comparison of percentage of indels sequenced (Y-axis) versus the locations of indels with respect to the double stranded break (DSB) site within the target region. Zero on X-axis indicates the location of the primer probe during sequencing. The number of WT, AsCas12a, and SpCas9 reads equals 7566750, 5605620, and 6010501, respectively. **C** Bar graph comparing the number of indels generated in WT and human TRAC gene (exon 2). Indels generated by WT and TRAC KO were ~0.3% and 0.04% overall respectively. Zero on X-axis indicates the DSB site. There was no appreciable difference between the number of WT and TRAC indels using either Cas12a or Cas9 enzymes. **D** shows off-targeting potential of CRAFT crRNA sequence revealing no exonic off-target matches (mismatch n = 4). **E** Flow cytometric data shows TCR and CD3 protein expression on primary T-cell surface after TRAC gene only KO, TRBC gene only KO (via CRAFT crRNA), and TRAC + TRBC multiplexed gene KO (via CRAFT crRNA).

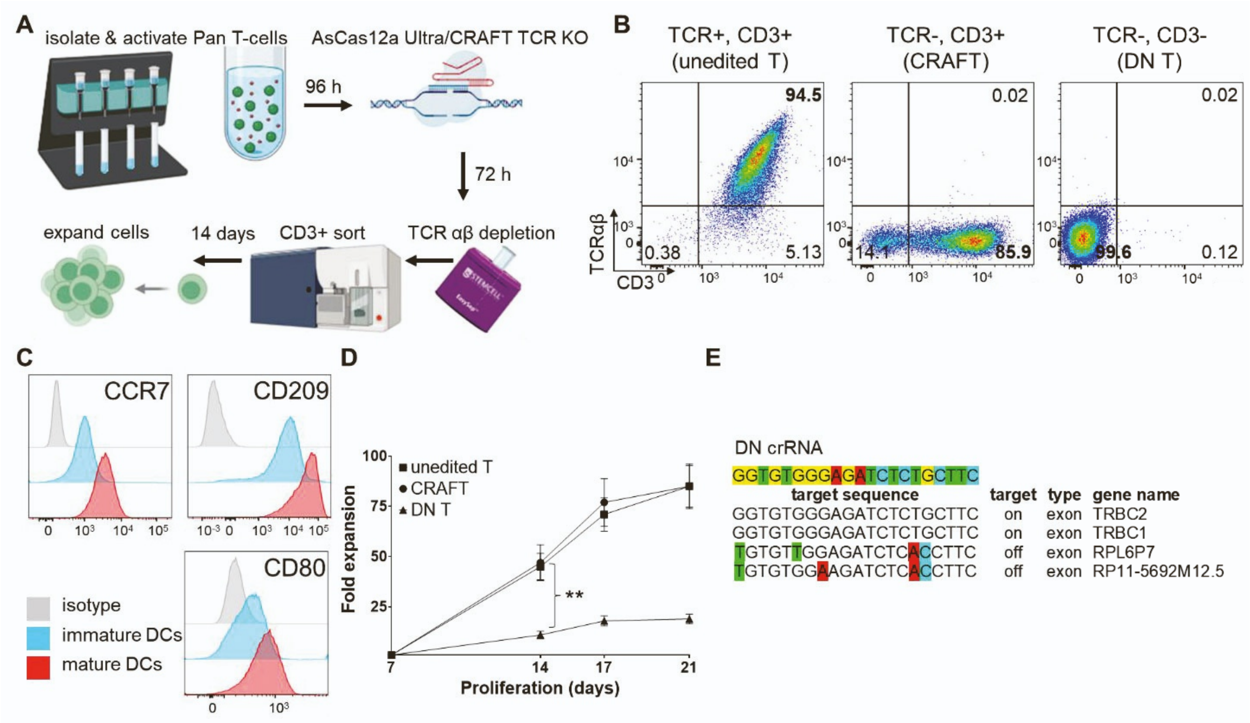


**Figure S2. Generation of CRAFT-cells and comparative ex vivo fold expansion to unedited T-cells, *related to Figure 2*.** **A** Schema of the timeline to generate, enrich, and expand CRAFT-cells derived from a healthy donor. The illustration was created with BioRender.com. **B** Representative flow cytometry dot plots of unedited T-cells, CRAFT-cells and TCR and CD3 double negative (DN) T-cells after expansion. **C** Flow cytometry panels showing DCs maturation markers (CCR7, CD209 and CD80) on day 9. Immature DCs did not receive polarizing agents (TNF-α, IL-1β, IL-6, and PGE2). Relevant isotype antibodies were used as controls. **D** The fold-expansion rate of unedited T-cells, CRAFT-cells, and DN T-cells up to 21 days. Mean ± SEM are plotted; multiple t-test indicating statistics on day 14 **, P<0.01. (n = 6). **E** shows off-targeting potential of TRBC (DN) crRNA sequence revealing two exonic off-target matches and the genes that would be disrupted. This guide RNA sequence was used to generate DN T-cells. The figure excludes multiple non-coding off-target candidates for the sake of conciseness and clarity.

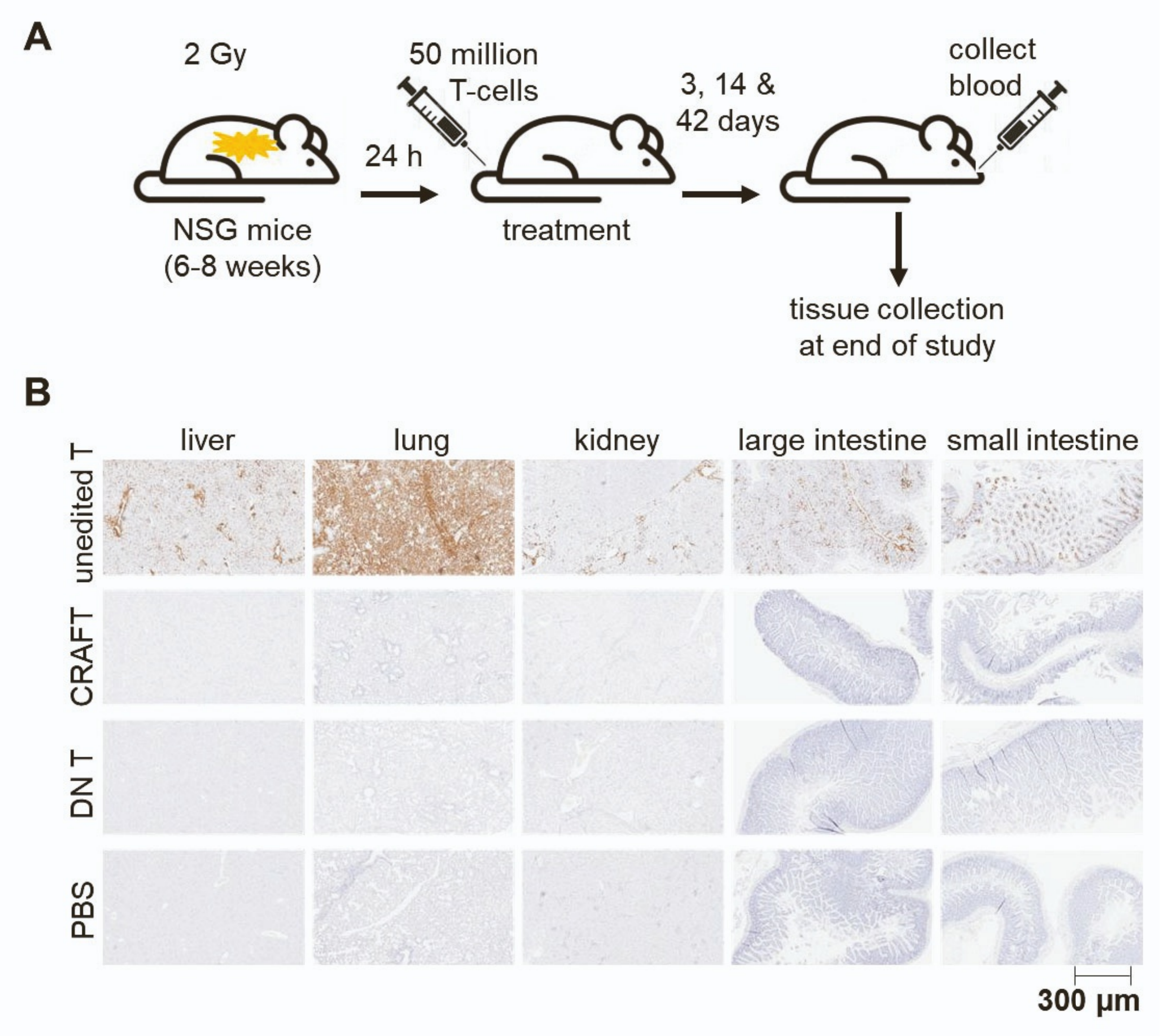


**Figure S3. CRAFT-cells do not cause GVHD in immunocompromised mice, *related to Figure 3*. A** Illustration of the experimental design to evaluate GVHD in NSG mice. NSG mice first received total body irradiation with a sublethal X-Ray dose of 2 Gy. 24 hours later irradiated mice were then engrafted with 50 million T-cells (treatment) of different phenotypes. Blood collections were performed on day 3, 14 and 42 after cellular treatment. Tissue for IHC was collected either at the end of study (for healthy mice) or when humane end point was reached (for moribund mice). **B** Representative immunohistochemical raw data show CD3+ cells per mouse, per study cohort (all tissues were from the same mouse per cohort). IHC was performed on all the animals that were included in various study replicates. The raw unmodified images were from the same experimental replicate. L-R: Liver, lung, kidney, large intestine, small intestine all images at 300 µm scale 10x magnification.

**A**

| sample name | cell no. (10^6) mean ±SD | viability (%) mean ±SD | EGFR+ (%) mean ±SD | TCR- (%) mean ±SD |
|---|---|---|---|---|
| **unedited CAR T** | 55.5 ±15 | 88.7 ±3 | 68.8 ±6 | 2.78 ±0.4 |
| **CRAFT CAR T** | 47.8 ±8 | 89.2 ±4 | 91.0 ±3 | 99.7 ±0.3 |
| **DN CAR T** | 58.5 ±21 | 89.7 ±5 | 70.6 ±4 | 99.8 ±0.3 |
| **non-CAR T** | 47.5 ±7 | 89.0 ±2 | 0.69 ±0.5 | 2.60 ±0.9 |

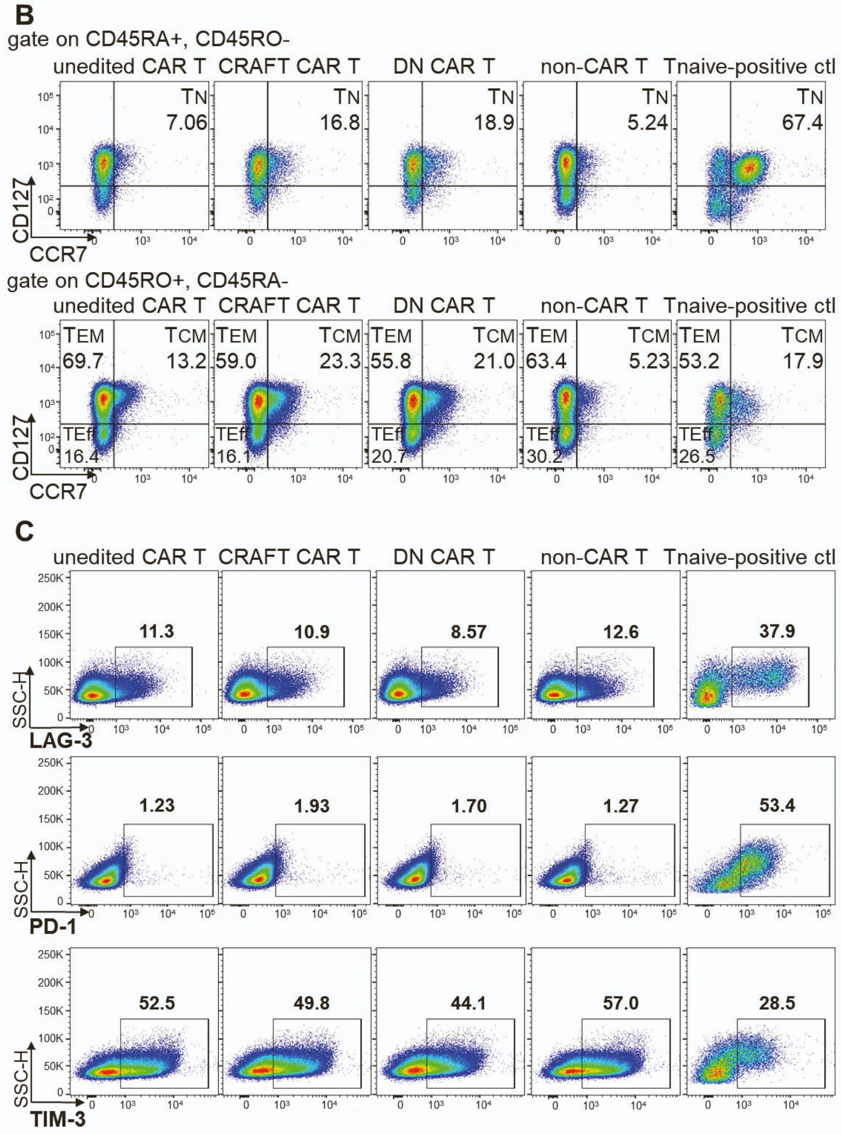


**Figure S4. Comparative growth rates of all allogeneic CAR T-cell fractions, *related to Figure 4*. A** Truncated EGFR incorporated in CAR construct doubles as a CAR potency marker as well as a safety switch. The mean values are reported (n = 4); error bars = ±SEM. **B** The phenotypic characterization of T-cell memory subsets was done using CD45RA and CD45RO. T-cells were further assigned memory subsets using CCR7 and CD127 within CD45RA+CD45RO– ($T_N$ cells) and CD45RA–CD45RO+ ($T_{CM}$, $T_{EM}$, and $T_{EFF}$ cells). The top plots depict $T_N$ cells (unlabeled quadrants are $T_{EMRA}$), while the bottom plots represent $T_{CM}$, $T_{EM}$, and $T_{EFF}$ cells. Naïve T-cells (CD14-, CD127+) were used as positive control. **C** Flow cytometric analysis comparing expression of common T-cell exhaustion markers (LAG-3, PD-1 and TIM-3) in CAR T-cell products on day 14 (final day of manufacture). T-cells re-stimulated with CD3/CD28 multiple times, were used to generate exhausted T-cell-like phenotype.

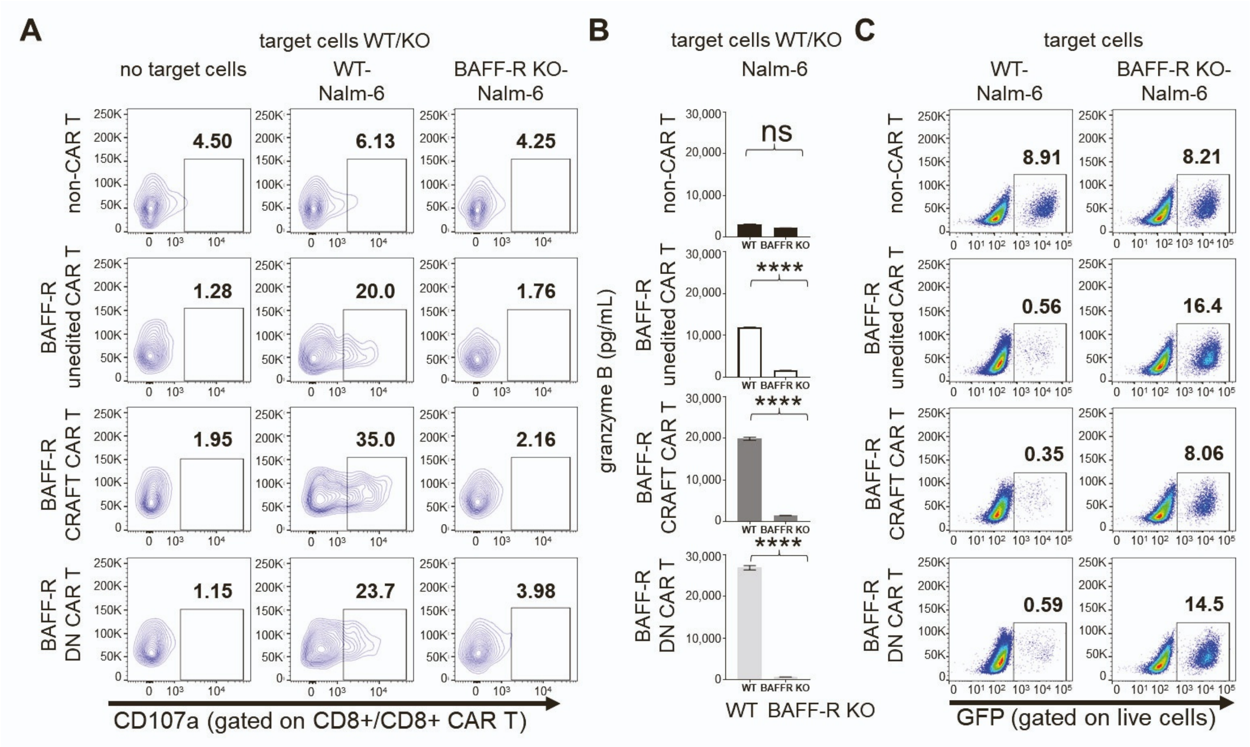


**Figure S5. Novel allogeneic CD8+ BAFF-R CAR T-cells show potent, antigen-specific cytotoxicity, *related to Figures 5*. A** Using CD107a degranulation assay, percentage of BAFF-R unedited CAR T-cells, BAFF-R CRAFT CAR T-cells, and BAFF-R DN CAR T-cells are shown to target BAFF-R Nalm-6 cells. The CAR T-cells were gated on CD8+ EGFR+, whereas non-CAR T-cells were gated on CD8+ alone (representative data included with total repeats of n = 3). **B** An ELISA showed granzyme B release (pg/mL) by the all the BAFF-R targeting CAR T-cells in response to Nalm-6 target cell lines. Non-CAR T-cells were a negative control (representative data shown for total repeats n = 3). Bar graphs plotted the means ± SEM. ****, P< 0.0001; ns, not significant. **C** The direct killing assay showed percent cytotoxicity of BAFF-R targeting CAR T-cells against Nalm-6-GFP cells. The non-CAR T-cells and the BAFF-R KO Nalm-6 target cells served as negative controls (representative data).

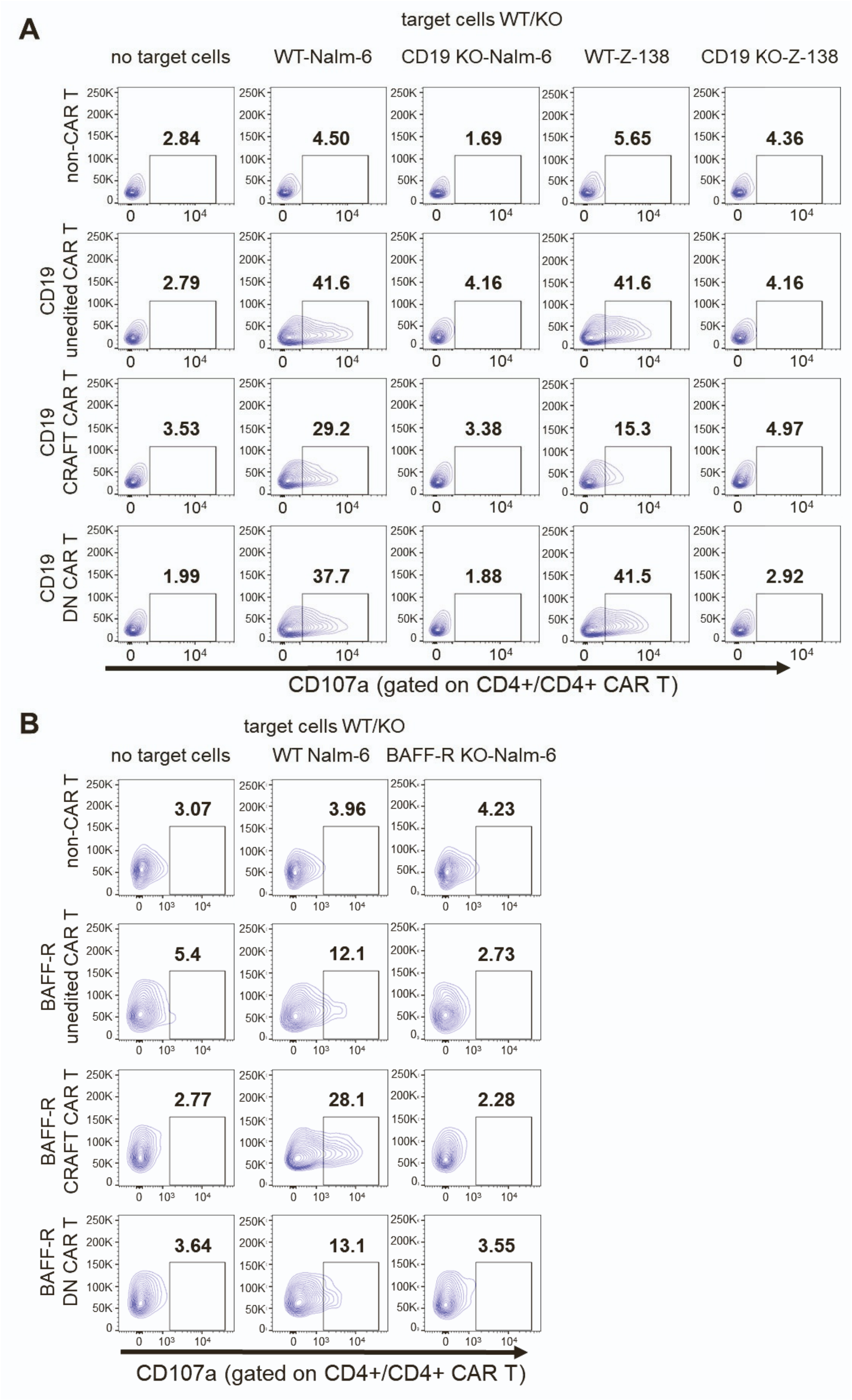


**Figure S6. Novel allogeneic CD4+ CD19 CRAFT CAR and CD4+ BAFF-R CRAFT CAR show potent, antigen-specific cytotoxicity.** ***Related to Figures 5.*** **A** CD107a degranulation assay shows various CD19 CAR T-cells target Nalm-6 and Z-138. The non-CAR T-cells served as a baseline/negative degranulation control. The CAR T-cells were gated on CD4+ EGFR+, whereas non-CAR T-cells were gated on CD4+ alone (representative figure, total repeats n = 3). **B** Similarly, CD107a degranulation assay using various BAFF-R CAR T-cells show targeting of Nalm-6 cells. (representative figure, total repeats n = 3).

**Table S1**. List of key material resources.

| REAGENT OR RESOURCE | SOURCE | IDENTIFIER |
|---|---|---|
| **Antibodies** | | |
| anti-human CD3ε clone UCHT1 BV605 | Biolegend | 300459 |
| anti-human TCR α/β clone IP26 APC/PE | Biolegend | 306718/306717 |
| Cetuximab biotin antibody | R&D Systems | FAB9577B |
| anti-human APC-streptavidin antibody | BD Biosciences | 554067 |
| SYTOX™ Blue Dead Cell Stain | Invitrogen | S34857 |
| SYTOX™ Green Ready Flow™ Reagent | Invitrogen | R37168 |
| anti-human CD14 clone M5E2 BV650 | BD Biosciences | 563419 |
| anti-human HLADR clone G46-6 PE | BD Biosciences | 555812 |
| anti-human CD11c clone SHCL-3 BV421 | BD Biosciences | 744434 |
| anti-human CD209 clone DCN46 APC | BD Biosciences | 551545 |
| anti-human CCR7 clone 2-L1-A BV786 | BD Biosciences | 566758 |
| anti-human CD80 clone B7-1 PerCP-Cy5.5A | BD Biosciences | 567437 |
| anti-human CD3 clone HIT3α BV605 | BD Biosciences | 564712 |
| anti-human CD4 clone SK3 PE-Cy7 | BD Biosciences | 557852 |
| anti-human CD8 clone SK1 APC-Cy7 | BD Biosciences | 348793 |
| anti-human EGFR AY13 clone BV421/APC | Biolegend | 352911/ 352906 |
| anti-human CD107a clone H4A3 APC | BD Biosciences | 560664 |
| anti-human CD45 clone HI30 PE/BUV395 | BD Biosciences | 555483/563792 |
| FxCycle™ Violet Stain (Fx Vio) | Invitrogen | F10347 |
| Propidium Iodide Ready Flow™ Reagent | Invitrogen | R37169 |
| **Cell lines and primary cells** | | |
| Nalm-6 luc-GFP (WT, KO CD19, KO BAFF-R) | [25] | - |
| Z-138 luc-GFP (WT, KO CD19) | [25] | - |
| PBMC cells from healthy donor | from clinic | - |
| Packaged Lentivirus (>4X 10^7 MOI= 3) | in house | - |
| **Critical commercial assays/ kits** | | |
| human pan T-cell isolation kit | Miltenyi Biotec | 130-096-535 |
| EasySep™ human TCR a/b depletion kit | Stemcell | 17847 |
| Human Granzyme B kit | Meso Scale Discovery | K151APDK |
| QIAamp DNA mini kit | Qiagen | 51304 |
| Click-iT™ EdU Cell Proliferation Kit | Thermo Fisher Scientific | C10337 |

| Recombinant proteins and chemicals | | |
|---|---|---|
| Recombinant human Interleukin-2/ Proleukin (aldesleukin) | Clinigen | NDC-76310-022-01 |
| Recombinant human Interleukin-15 | Celgeneix GMBH | 1013-050 |
| Recombinant human Interleukin-4 | PeproTech | 200-04 |
| Recombinant human GM-CSF | PeproTech | 300-03 |
| Recombinant human TNFα | PeproTech | 300-01A |
| Recombinant human Interleukin-1β | PeproTech | 200-01B |
| Recombinant human Interleukin-6 | PeproTech | 200-06 |
| PGE2 | Stemcell | 72192 |
| Blinatumomab (Blincyto) | from Clinic | - |
| X-VIVO™ 15 media | Lonza | 04-418Q |
| Head-inactivated human male AB plasma | Sigma-Aldrich | H3667 |
| Dynabeads Human T-Activator CD3/CD28 | Gibco | 11131D |
| CRISPR crRNAs and sgRNA | IDT | made-to-order |
| Alt-R™ A.s.Cas12a/Cpf1 Ultra nuclease | IDT | 10001273 |
| Alt-R® Cpf1 Electroporation Enhancer | IDT | 1076301 |
| Alt-R™ S.p. HiFi Cas9 Nuclease V3 | IDT | 1081060 |
| Alt-R® Cas9 Electroporation Enhancer | IDT | 1075915 |
| Nuclease-free 1X IDTE solution | IDT | 11-05-01-13 |
| P3 primary cell 4D 96-well nucleofector kit S | Lonza | PBP3-00675 |
| GolgiStop™ Protein Transport Inhibitor | BD Biosciences | 554724 |
| Diluent 3 | Meso Scale Discovery | R50AP |
| Diluent 58 | Meso Scale Discovery | R50CA |
| Wash buffer 20X | Meso Scale Discovery | R61AA |
| MSD gold Read buffer B | Meso Scale Discovery | R60AM |
| RPMI-1640® medium | ATCC | 30-2001 |
| Iscove's Modified Dulbecco's Medium (IMDM) | ATCC | 30-2005 |
| Fetal bovine serum (FBS) | Thermo Fisher Scientific | 16140071 |
| PBS-1X | Fisher | MT21040CV |
| Ficol-Paque™ | Cytiva | 45-001-751 |
| ACK lysis buffer | Gibco | A1049201 |
| MACS® LS columns | Miltenyi Biotec | 130-042-401 |
| Heparin tubes | Sarstedt | 20.1345.100 |
