# Supplementary material for: Novel allogeneic CAR T-cell platform involving microhomology-mediated end joining repair and low off-targeting potential
Source: Mol Ther Nucleic Acids. 2025 Nov 17;36(4):102778. doi: 10.1016/j.omtn.2025.102778 (PMC12704299; doi:10.1016/j.omtn.2025.102778)
Supplement: Document S2. Article plus supplemental information [file mmc2.pdf]

Molecular Therapy
Nucleic Acids
Original Article

Novel allogeneic CAR T-cell platform involving microhomology-mediated end joining repair and low off-targeting potential

Tanya Hundal,[1] Yan Luo,[1,2] Yaqing Qie,[1] Martha E. Gadd,[1] Andrew D. Brim,[1] Isas Vazquez-Rosario,[1] Shaohua Guo,[1] Mohamed A. Kharfan-Dabaja,[3,4] and Hong Qin[1,2,4,5]

[1]Regenerative Immunotherapy and CAR-T Translational Research Program, Mayo Clinic, Jacksonville, FL, USA; [2]Department of Cancer Biology, Mayo Clinic, Jacksonville, FL, USA; [3]Division of Hematology and Medical Oncology, Department of Internal Medicine, Mayo Clinic, Jacksonville, FL, USA; [4]Blood and Marrow Transplantation and Cellular Therapy Program, Mayo Clinic, Jacksonville, FL, USA; [5]Department of Immunology, Mayo Clinic, Rochester, MN, USA

Several allogeneic chimeric antigen receptor (CAR) T-cell therapies in clinical trials rely on CRISPR-Cas genome editing, but the enzyme's random repair mechanism increases the risk of undesired off-target effects, challenging safe CAR T-cell generation. To address this, we developed a novel CRISPR RNA (crRNA) targeting the T-cell receptor beta constant (TRBC) gene. Combined with AsCas12a Ultra, this crRNA edits primary human T-cells via a predictable microhomology-mediated end joining (MMEJ) DNA repair pathway, significantly lowering off-target risks. During evaluation, we sequestered a unique T-cell subset with disrupted T-cell receptor (TCR), retained CD3 expression, and no *in vivo* alloreactivity. Termed CD3-retained, allogeneically functioning T-cells (CRAFT-cells), these cells exhibited growth kinetics comparable to unedited T-cells. When engineered with CD19- or BAFF-R-targeted CARs, CRAFT CAR T-cells showed strong antigen-specific cytotoxicity and significant *ex vivo* expansion compared to conventional CD3-disrupted CAR T-cells. Moreover, CRAFT CAR T-cells effectively served as effector cells for bispecific T-cell engagers (BiTEs), enabling CD3-dependent tumor cell killing. Our CRAFT crRNA platform offers a novel strategy to generate safer allogeneic CAR T-cells. The distinct properties of CRAFT CAR T-cells, combined with BiTE therapy, represent a promising and potentially more durable approach for next-generation allogeneic CAR T-cell therapies in clinical applications.

INTRODUCTION

Allogeneic chimeric antigen receptor (CAR) T-cell therapy, as opposed to the individualized autologous CAR T approach, offers the benefits of using a readily available, higher quality and quantity of starting donor T-cells.[1] Since allogeneic CAR T-cells have the potential to generate a large number of doses from a single manufacturing process, allogeneic CAR T-cells are coveted as a promising "off-the-shelf" therapy.[2] However, acute T-cell causative toxicity may occur when alloreaction targets the host tissues, specifically directed against the host's major histocompatibility complex (MHC) that is mediated via T-cell receptor (TCR) on the donor CAR T-cells.[3] The risk of life-threatening graft-versus-host disease (GVHD), where donor TCR can mount an immune offensive against the alloantigens presented by the recipient cells, can be mitigated by knockout (KO) of the donor TCR.[4]

The TCR-CD3 is an octameric assembly with the core dimer of TCRαβ, which is supported at the T-cell surface with the additional dimers of CD3δε, CD3γε, and CD3ζζ.[5] TCRαβ is the antigen recognition element with both subunits having extracellular domains with distal antigen recognizing variable regions and membrane proximal constant regions. The relatively conserved constant regions also contain transmembrane domains (TMs) that stabilize the entire complex.[6] Thus, targeting the TCR to generate an allogeneic T-cell therapy is a common strategy that focuses on targeting either the constant region of TCR α (via *TRAC* KO) or the constant regions of both TCR α and TCR β (*TRBC*), with many of the resulting allogeneic T-cells advancing into clinical trials.[7] To date, there are a limited number of phase 1 clinical trials evaluating allogeneic CAR T therapies that were generated by editing *TRBC* only, either via CRISPR-SpCas9 or other legacy genome editing tools.[8,9] Unfortunately, CRISPR-SpCas9-mediated, *TRBC* KO-generated allogeneic CAR T-cells have reported a lack of persistence in pre-clinical models, suggesting the necessity of greater understanding and innovation.[10]

One key issue encountered during CRISPR-Cas genome editing is the plethora of insertion and deletion (indel) sequences generated at the target DNA break site. This randomness in the sequence repair outcomes is due to the activation of canonical non-homologous end-joining (NHEJ) pathway, which can cause the majority of the alleles

Received 25 June 2025; accepted 10 November 2025;
https://doi.org/10.1016/j.omtn.2025.102778.

Correspondence: Hong Qin, MD, PhD, Associate Professor of Medicine, Division of Hematology/Oncology Director, Regenerative Immunotherapy & CAR-T Translational Research Program Mayo Clinic, Florida 4500 San Pablo Rd S, Jacksonville, FL 32224, USA.
E-mail: qin.hong@mayo.edu

© 2025 The Author(s).
Published by Elsevier Inc. on behalf of The American Society of Gene and Cell Therapy.
This is an open access article under the CC BY license (http://creativecommons.org/licenses/by/4.0/).

to remain in-frame; thus, the desired frameshift alleles in KO cells continue to remain a minority outcome.[11,12] To counter this, we strategically improved the sequence homogeneity by preemptively designing a crRNA that engages microhomology-mediated end joining (MMEJ) DNA repair pathway. Herein, short homologous sequences across the DNA cut-site help to seal the break, thus biasing toward an improved precision repair.

SpCas9 enzyme has been widely adopted in the clinical setting and has been used exhaustively in allogeneic CAR T-cell manufacture for several programs.[13–15] However, SpCas9 is notorious for poor target site specificity causing liberal off-target mismatches.[16] Mismatch to the unintended regions can lead to an increase in mutational burden and heighten the risk of malignancy.[17] Specifically, SpCas9 has been known to cause larger structural variations in primary human T-cells, due to the haphazard repair of the blunt-ended DNA break site, primarily via NHEJ.[18] Alternatively, AsCas12a enzyme has a higher targeting fidelity due to its shorter size, more stringent CRISPR RNA (crRNA) requirement, and the ability to cut DNA strands in a staggered fashion to promote MMEJ. However, wild-type (WT) AsCas12a enzyme has a low editing efficiency, whereas AsCas12a Ultra, the most recent Cas12a iteration, has editing efficiencies approaching 100% in primary human cells, with minimal off-target risk during multiplex genome editing.[19,20] Due to its recent discovery, AsCas12a Ultra is yet to be adopted into allogeneic clinical trials.

In our current research, we use MMEJ repair-inducing TRBC crRNA with AsCas12a Ultra to edit TCR for the generation of allogeneic CAR T-cell therapy. We demonstrate that our crRNA selectively instigated MMEJ repair pathway and significantly reduced any off-target mismatches to meaningfully lower the genotoxic burden. Interestingly, we also report the discovery of a novel CD3-positive cell population with dysfunctional TCR (CD3-retained, allogeneically functioning T-cells [CRAFT]-cells), which when enriched and tested, demonstrated no *in vivo* alloreactivity. Using these novel CRAFT-cells, we then manufactured and characterized allogeneic CRAFT CAR T-cells that showed growth, potency, and cytotoxic function on par with the unedited CAR T-cells. In addition, CRAFT CAR T-cells can further enhance killing of leukemia cells that are localized via bispecific antibody engagement. The discovery of this distinctive CRAFT-cell population heralds the ability to decouple TCR binding from CD3 signaling. While CD3 continued to remain intact on the CRAFT-cell surface, this modular CRAFT CAR T-cell population can be included as a part of a novel allogeneic CAR T-cell product. Such allogeneic CAR T-cells can then potentially be used with bispecific antibodies for consolidated treatment in immunocompromised patients.

RESULTS

Unique crRNA mediates an efficient TCR β-specific knockout to generate allogeneic T-cells with MMEJ repair and low off-targeting

We adopted a strategy for the generation of allogeneic T-cells by targeting the constant region of TCR β (TRBC) and the evaluation of the efficiency of TCR genomic editing. To compare the editing efficiencies, we devised two TRBC editing strategies using (1) a CRAFT crRNA (hereafter labeled as crRNA) designed to be compatible with AsCas12a Ultra or (2) a single guide RNA (sgRNA) that worked in tandem with SpCas9 to generate ribonucleoprotein (RNP) complexes. Although KO evaluation revealed 98%–99% abrogation of TCR protein using either of the CRISPR-Cas strategies (Figures 1A and S1A), deep amplicon sequencing of TCR genes revealed that the site-specific KO with the AsCas12a Ultra/CRAFT crRNA duo achieved 95.4% editing efficiency, while the SpCas9/sgRNA pair achieved significantly less overall editing at 64.6% (Figure 1B). The crRNA approach produced greater site-specific indels, with an incredibly low insertional rate (0.1%) versus 4% insertions with SpCas9/sgRNA (Figure S1B). The percent on-target, on-position deletion rate via crRNA is 90.6% as opposed to the rate of 44.7% with the sgRNA approach (Figure S1B). Moreover, the plateauing observed at the target region with sgRNA further highlights indiscriminate on-target, off-position deletions (Figure S1B). As noted in Figure 1B, using CRAFT crRNA significantly lowered the on-target, off-position deletions to 4.6% compared to SpCas9/sgRNA with 15.9%. Importantly, by using CRAFT crRNA, the topmost indel generated was via an MMEJ-guided repair initiation (Figure 1C, bottom), whereas the top indel hit with sgRNA was a WT/substitution, (Figure 1C, top) suggesting a reason for 35.4% of the genome remaining unedited with the SpCas9/sgRNA duo.

While TRBC locus was targeted, the constant region of TCR α (TRAC) gene remained untouched (Figure S1C) using either editing method. The staggered cuts utilized by AsCas12a Ultra also resulted in the on-position repair of 11% of the genome via MMEJ, a pathway that was distinctly absent from the SpCas9 strategy. We used two *in silico* off-target prediction tools, CRISPR-Cas9 Target online predictor (CCTop) and Cas-OFFinder, to query the potential off-target sites for specific gRNAs used. The TRBC sgRNA had the potential to sever six other protein-coding sites (exons), targeting tumor suppressors like WNT3A (Figure 1D).[21] Additionally, detrimental off-targeting to DENND1B can result in a defective TCR internalization machinery, causing abnormal T-cell effector functions like asthma.[22] The uniqueness of CRAFT crRNA is further demonstrated by the negligible off-target assaults with none attacking the exons of another random gene (Figure S1D). Overall, we observed CRAFT crRNA elicited *in situ* repair, circumventing NHEJ in the process, and significantly reduced off-targeting rate in all TCR-abrogated cells.

Further characterization of protein KO using either of the gene editing strategies revealed the abrogation of TCR and, surprisingly, changes in the surface expression of CD3 that resulted in two distinct T-–cell populations: a major population (85%–90%) where CD3 expression is absent and a minor population (10%–15%) where CD3 remained surface intact even after TCR KO (Figures 1E and S1B). This minor population of edited T-cells was named CRAFT-cells and was only observed when each TRBC1 and TRBC2 gene was edited at exon 1, in the region between N91 and P115 residues.

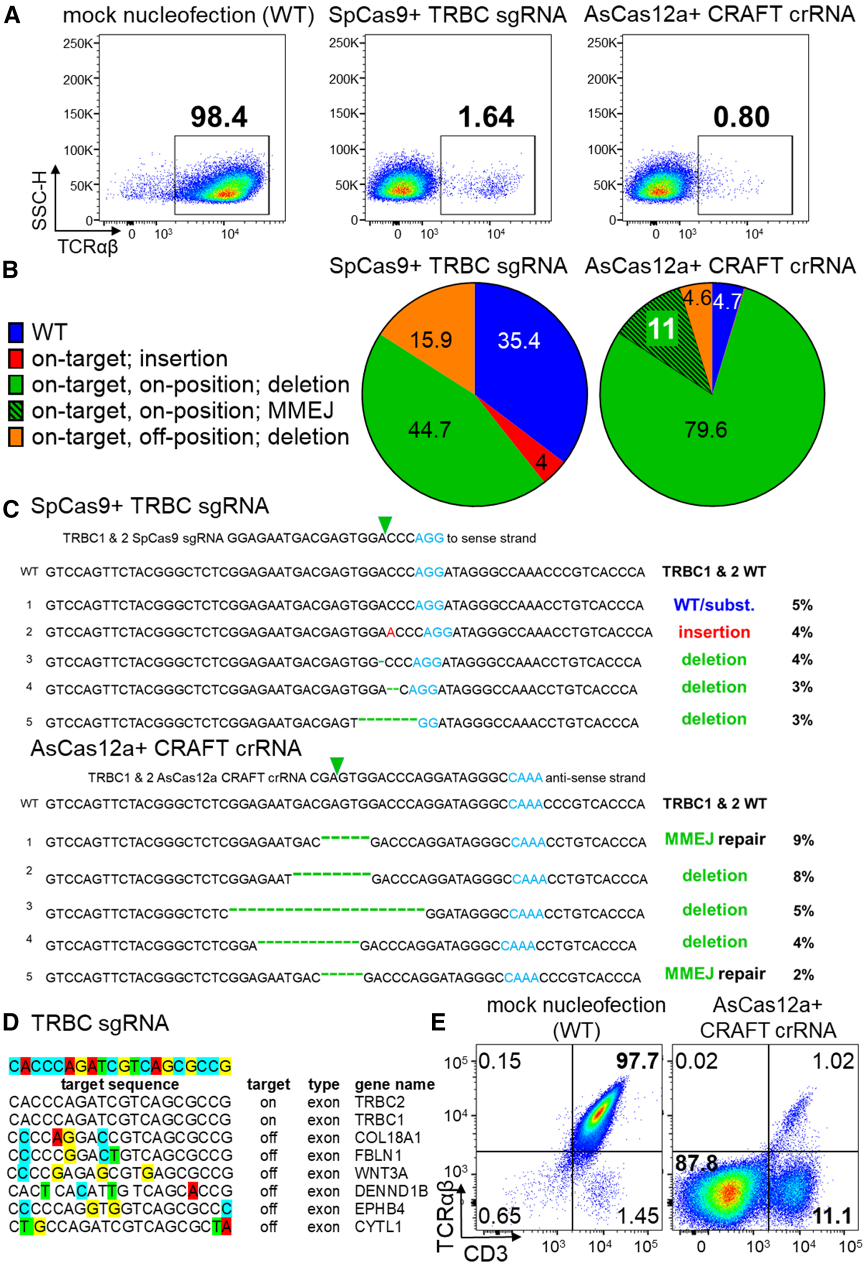

Figure 1. Unique crRNA mediates an efficient TCR β-specific knockout to generate allogeneic T-cells with MMEJ repair and low off-targeting

(A) Flow cytometric data describing percent surface-level protein expression of TCR αβ. AsCas12a Ultra coupled with TRBC crRNA (termed as CRAFT crRNA) and SpCas9 enzyme with TRBC sgRNA were utilized to edit primary human T-cells. (B) Pie graphs show percentage of outcomes generated after KO using SpCas9/TRBC sgRNA pair versus using AsCas12a Ultra/CRAFT crRNA pair. Blue = % WT (unedited genome); red = % insertion during indel repair; green = % on-target, on-position deletion; black hatch pattern on green = % on-target, on-position MMEJ DNA repair; orange = % on-target, off-position deletion. On-target = on the target gene of interest, on-position = double-stranded break (DSB) on the predicted nucleotide at the target gene. (C) Top five locus-specific indel types and ratios in *TRBC* after NGS amplicon sequencing (Top: SpCas9/TRBC sgRNA pair; bottom: AsCas12a/CRAFT crRNA pair). AsCas12a/CRAFT crRNA pair targeted the *TRBC1* and *TRBC2* loci via MMEJ. The sgRNA and anti-sense crRNA sequences are aligned at the top of each panel to highlight corresponding site-specific indels. The top sequences account for 19% of all indels generated with TRBC sgRNA and 28% of indels with CRAFT crRNA. Each nucleotide deletion is indicated by (–). Inverted triangles indicate the location of primary DSB site, PAM indicates protospacer-adjacent motif (TTTV for AsCas12a and NGG for SpCas9). (D) The TRBC sgRNA off-target sequence comparison revealed six exonic off-target matches that would be disrupted. The figure excludes multiple intronic and intergenic (non-coding) off-target candidates for the sake of conciseness and clarity. (E) Flow cytometry data exploring percent TCR and CD3 surface protein expression after AsCas12a Ultra and CRAFT crRNA is used to KO TCR β in primary human T-cells. This single activity generated an expected TCR−, CD3− (DN) population of ~88% and a unique TCR−, CD3+ population ~11% (termed as CRAFT-cells). Also see Figure S1 for additional details.

TCR editing in the exonic regions outside of this zone resulted in the simultaneous disruption of CD3 expression from T-cell surface. Additionally, flow cytometric data in Figure S1E compare the corresponding TCR and CD3 protein expression on T-cells after (1) TRAC gene KO, (2) TRBC gene KO (via CRAFT crRNA), or (3) multiplexed TRAC + TRBC gene KO (via CRAFT crRNA). The data indicate that gene editing to target TRAC gene alone did not produce any CRAFT-cell population, and that CRAFT-cells are lost whenever TRAC gene is disrupted. These data, taken together with the *TRAC* remaining untouched during amplicon sequencing of TRBC only KO cells, indicate that the TCRα protein is a critical part of CRAFT-cell make-up.

CRAFT-cells evade alloreactivity *in vitro*

The concomitant loss of CD3 surface expression with TCR KO and the inability of the resulting double-negative T-cells (DN T) to cause alloreactivity *in vitro* has been reported previously.[4,10] However, the identification of a TCR KO that retained CD3 surface expression was novel and spurred our investigation of alloreactivity of this CRAFT crRNA-generated CRAFT-cell population. To do so, we

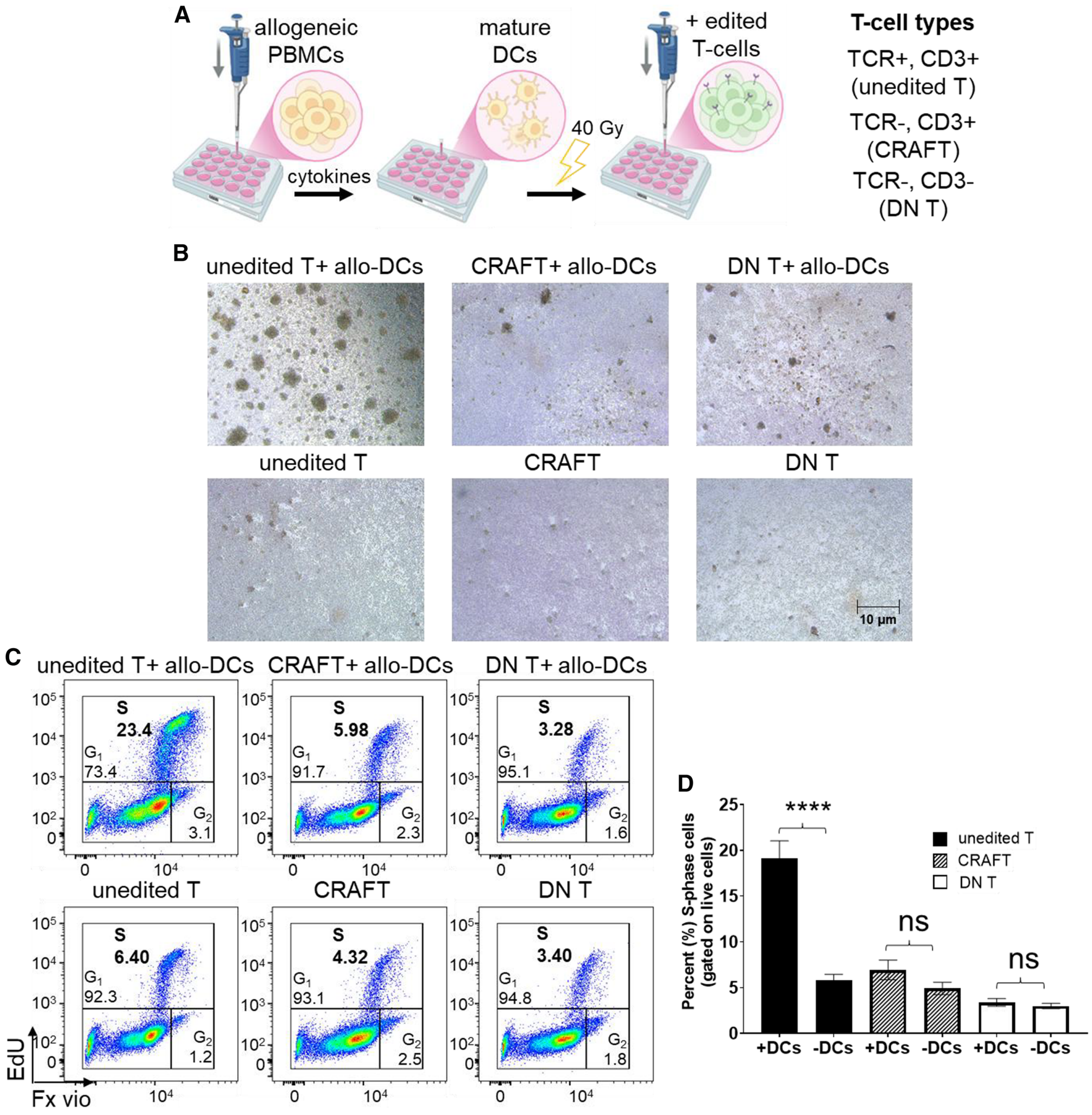

Figure 2. Newly identified CRAFT-cells evade alloreactivity *in vitro*

(A) Illustration of experimental layout to assess if allogeneic T-cells (either CRAFT- or DN T-cell fractions) could cause alloreactivity against mature DCs derived from multiple different donor unmatched PBMCs. Unedited T-cells were kept as positive control for alloreactivity. Cryopreserved unedited T-cells, CRAFT-cells, and DN T-cells that were generated from T-cells of a different/unmatching donor were co-cultured with irradiated allogeneic DCs in at a DC:T ratio of 1:10, for 96 h. The illustration was created with BioRender.com. (B) Representative images showing mixed lymphocyte reaction of enriched, edited T-cells with irradiated allogeneic DCs. Images were acquired on Leica light microscope after 96 h of incubation (scale bar, 10 μm). The images are from the same experimental replicate. (C) The percentage of live T-cells entering S-phase was determined via an EdU dye incorporation assay. Fx Vio = Fx cycle violet DNA stain. (D) Bar graph representing aggregated data showing the percentage of live T-cells entering S-phase. Replicated experiments using different T-cell cohorts (n = 10) were performed and compared to a control without DCs; the means and associated SEM were plotted. ****p < 0.0001; ns, not significant. Also refer to Figure S2 for CRAFT-cell growth data.

developed a protocol to sort and expand CRAFT-cells (Figure S2A) and evaluated reactivity against irradiated allogeneic multi donor-derived mature dendritic cells (allo-DCs) in a mixed lymphocytic reaction, as illustrated in Figure 2A. Unedited T-cells and enriched DN T-cells were used as alloreactivity-positive and -negative controls, respectively (Figure S2B). To maintain the uniformity in the editing methods, both CRAFT-cell and DN T-cell fractions were generated using AsCas12a Ultra enzyme. After co-incubation of the stimulator allo-DCs (Figure S2C) with the different responder T-cells for 4–5 days, various T-cell phenotypes were evaluated by light microscopy to observe proliferative changes (Figure 2B). T-cell division was quantified using an 5-ethynyl-2′-deoxyuridine assay (EdU) incorporation flow cytometry assay; upon interacting with the allo-DCs, the responder cells were activated and entered DNA replicating S-phase

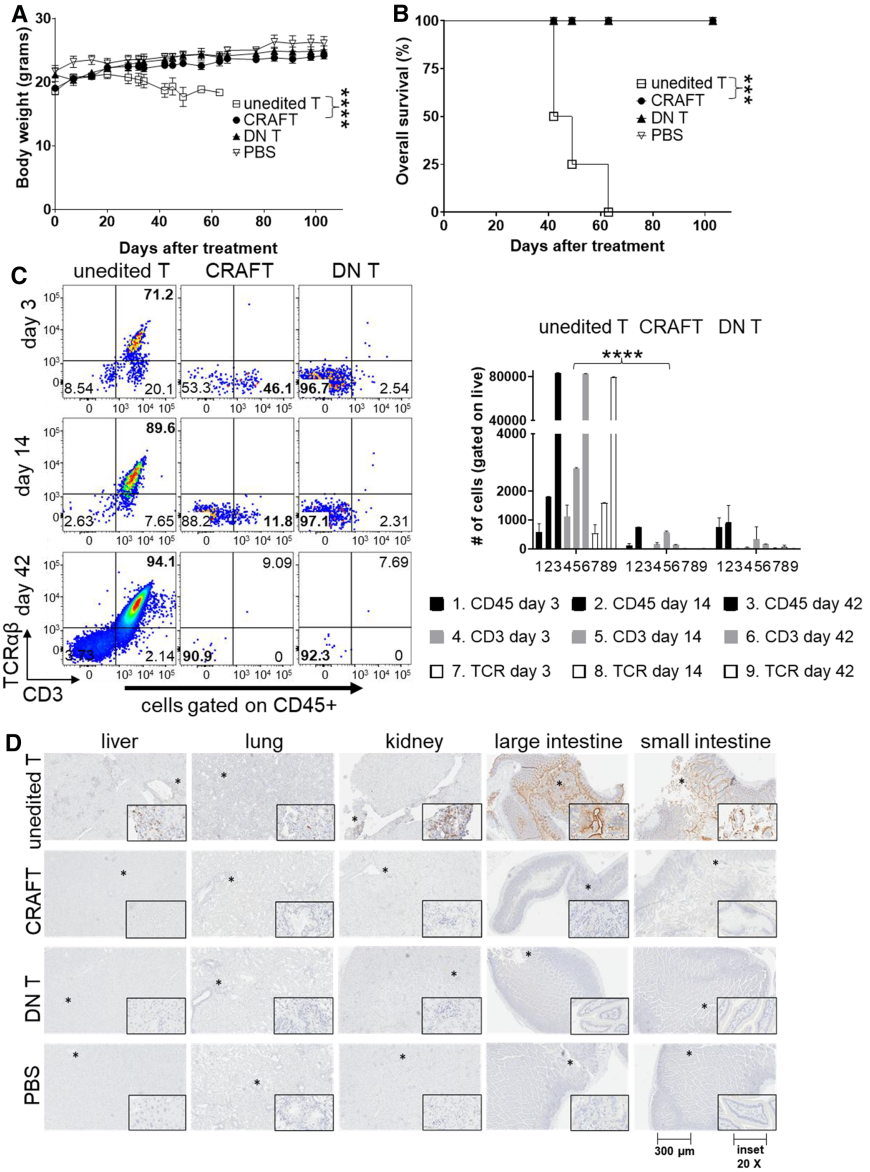

Figure 3. CRAFT-cells do not cause GVHD in an immunodeficient mouse model

(A) NSG mice received total-body irradiation using a sublethal dose of 2 Gy; 1 day later, mice ($N = 4$) received a treatment of either PBS or 50 million gene T-cells, namely, unedited T-cells, CRAFT-cells, or DN T-cells. A representative plot of the mean body weight (grams) after treatment shows GVHD-related deaths of the mice receiving unedited T-cells compared to all other treatments. The experiment was repeated to allow a $n = 18$ for each treatment cohort. ****$p < 0.0001$ by Mann-Whitney test. Loss of error bars indicated a surviving single animal. (B) Representative Kaplan-Meier plot of overall survival data from (A). Log rank test: ***$p < 0.001$. (C) Blood collected from mice on days 3, 14, and 42 was evaluated as percent of human T-cells by gating on CD45+ cells. Numbers (1–9) in the x axis correspond to each bar graph in the figure, and the descriptors are arranged sequentially below the graph. In this representative figure, the CD3+TCR+ T-cells have been retained on day 42; however, the CRAFT-cells (TCR−, CD3+) and DN T-cells (TCR−, CD3−) are absent by day 42. The bar graph summarized the absolute number of engrafted T-cells at days 3, 14, and 42 by detecting surface expression of CD45 (black bars), CD3 (gray bars), and TCR (white bars). The population is gated on live cells. Graph was plotted using mean with SEM. ****$p < 0.0001$. (D) Representative immunohistochemical raw data show human CD8α+ cells infiltrating various organs of a mouse receiving unedited T-cells (all tissues were from the same mouse per cohort); all other T-cell-treated mice had undetectable staining for human CD8α+ cells. Immunohistochemistry (IHC) was performed on all the animals that were included in various study replicates. The raw unmodified images are from the same experimental replicate. Left to right: liver, lung, kidney, large intestine, small intestine; all images at 300 μm scale and 10× magnification (inset = 20×). Asterisk symbols indicate the magnified area in the inset figure. Also refer to Figure S3 for IHC.

thus incorporating EdU. As seen in Figures 2C and 2D CRAFT-cells incorporated significantly lower amount of EdU as compared to the unedited T-cells. Upon stimulation with anti-CD3/CD28 beads and monitoring the cells for 14 days of expansion, CRAFT-cells and unedited T-cells grew with a similar fold expansion rate, whereas DN T-cells responded significantly slower to the CD3/CD28 restimulation (Figure S2D). To summarize, CRAFT-cells did not elicit an alloresponse, while still being able to be stimulated and grow at the rate similar to unedited T-cells.

CRAFT-cells do not cause GVHD in immunodeficient mice

With complete TCR abrogation, traditional allogeneic DN T-cells do not cause GVHD in immunodeficient animals, such as – (NSG) mice.[4,23] To evaluate CRAFT-cells as the starting material for a future allogeneic CAR T-cell therapy, we injected 50 million gene-edited T-cells into sublethally irradiated NSG mice and monitored them for clinical signs of GVHD that included weight loss, hunching, and alopecia (Figure S3A). The unedited T-cells served as positive control that caused GVHD-induced weight loss and subsequent mortality of mice within 70 days (Figures 3A and 3B). Meanwhile, the animal cohorts that received enriched CRAFT- or DN T-cells did not show any physical signs of GVHD and continued to survive even after the end of study (100 days). During the study, blood was 2drawn on days 3, 14, and 42 and evaluated for the presence of CD45-positive human T-cells (Figure 3C, bar graph). The unedited T-cells continued to remain engrafted and proliferated, whereas

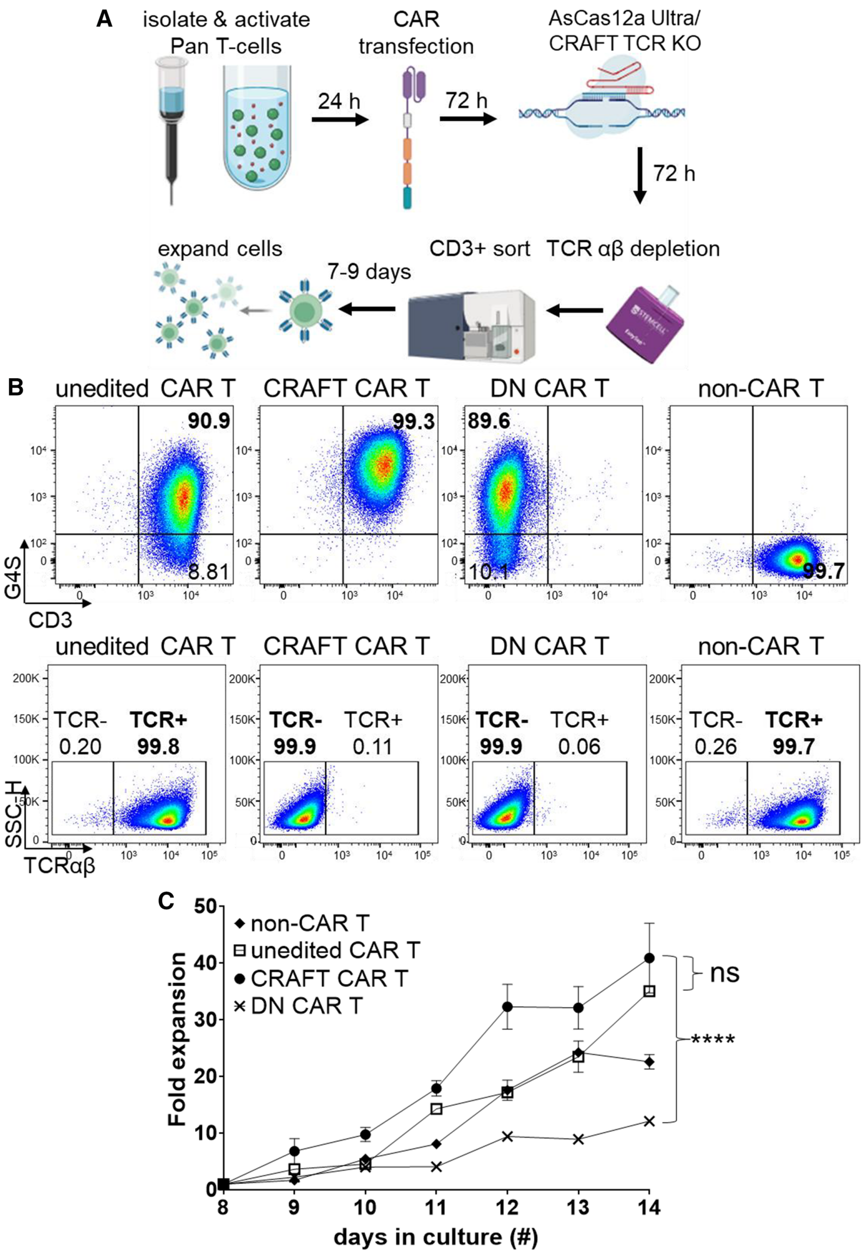

Figure 4. Generation and characterization of novel allogeneic CRAFT CAR T-cells

(A) A schema for generating allogeneic CRAFT CAR T-cells. Pan T-cells were isolated from healthy donor PBMCs and were activated using CD3/CD28 paramagnetic beads overnight before transducing with CAR lentivirus. T-cells were edited with AsCas12a Ultra using CRAFT or DN crRNA. After protein turnover, TCR+ T-cells were depleted, and the remaining TCR-negative cells were sorted into CD3+ and CD3− populations and expanded for 7–9 days. The illustration was created with BioRender.com. (B) Flow cytometry data comparing the CAR potency percent (G4S, top panel) and percent identity as determined by the combination of CD3 (top panel) and TCR (bottom panel) of unedited CAR T-, CRAFT CAR T-, DN CAR T-cells, and non-CAR T-cells were determined on the final day of manufacture (representative data n = 2, different donors). (C) Normalized fold expansion of CRAFT CAR T-cells to unedited CAR T- and DN CAR T-cells from days 8 to 14, after sorting all the cells on day 6 and stimulating all controls with CD3/CD28 beads (1:1) for 48 h (representative data n = 2, different donors). All data have been normalized to day 8 after de-beading the cells. The sorted cells were expanded using cytokines only. The mean values are reported; error bars = ±SEM. Multiple t test indicating statistics on day 14. ****p < 0.0001 between CRAFT CAR T- and DN CAR T-cells. Also refer to Figure S4 for additional CAR T-cell characterization.

CRAFT- and DN T-cells lost their engraftment after 2 weeks. The CD45+ engrafted cells were further evaluated for CD3 and TCR expression to determine the phenotype of the different T-cells in the treatment cohorts (Figure 3C). By day 42, the cohort that received unedited T-cells was moribund, and the analysis of blood from these mice largely contained CD45+, CD3+, TCR+ T-cells. In contrast, the mouse cohorts that were treated with either CRAFT- or DN T-cells remained healthy and did not retain any TCR+ T-cells at day 42. At the end of the study, the CRAFT and DN T mouse cohorts were euthanized, and vital organs were collected and immunohistochemically evaluated for residual CD8α- and CD3-positive T-cells (Figures 3D and S3B, respectively). The absence of infiltrating T-cells confirms the lack of alloreactivity of CRAFT-cells and the reduced potential to cause GVHD 22*in vivo*.

Validating CRAFT-cells as a viable platform for allogeneic CAR T-cell production

We were inspired by several publications that enumerated their methodologies to generate allogeneic CAR T-cells.[4,10,23] Following those, we developed a strategy to generate two types of allogeneic CAR T-cells in which TCR was edited after CAR transfection, followed by a modified gating strategy to sort either CRAFT CAR T-cells or DN CAR T-cells (Figure 4A). T-cells were transduced using CAR lentivirus that either targeted CD19 or B-cell-activating factor receptor (BAFF-R). The cells were then edited using AsCas12a Ultra and the appropriate crRNA to either produce DN CAR T-cells or CRAFT CAR T-cells. Following the sort of CD3+ edited CAR T-cells, the CRAFT CAR T-cells were expanded using only signal-3-conducive cytokine support. At the end of the expansion phase, the identity of the edited CAR T-cells was established via both CD3 and TCR antibodies, while CAR potency was established with the anti-G4S antibody against the G4S linker in CAR sequence (Figure 4B). CRAFT CAR T-cells had a significantly higher *ex vivo* fold expansion compared to DN CAR T-cells and a growth rate

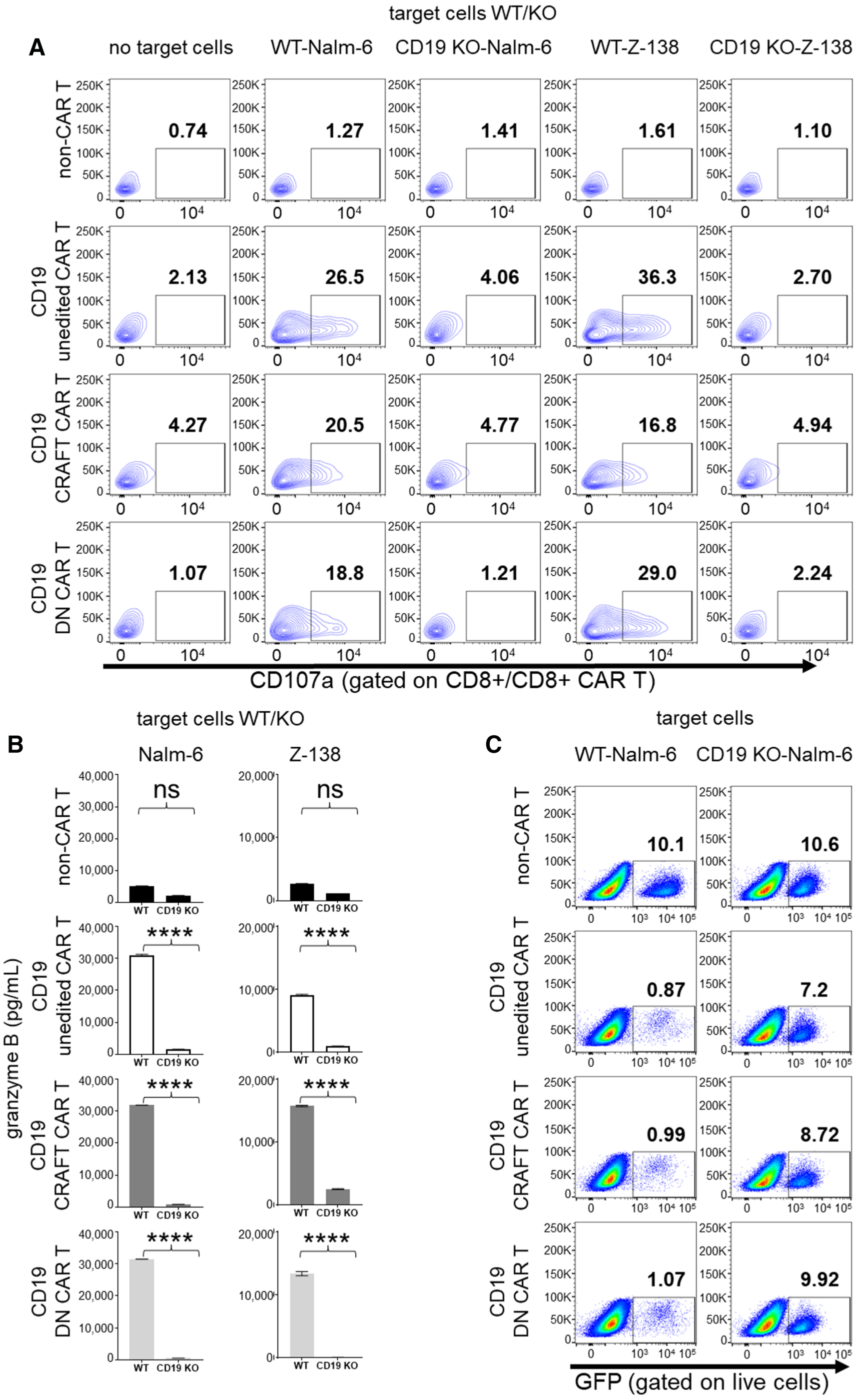

Figure 5. Novel allogeneic CD19 CRAFT CAR T-cells show potent, *in vitro* antigen-specific cytotoxicity

(A) Using CD107a degranulation assay, percentage of CD19 unedited CAR T-cells, CD19 CRAFT CAR T-cells, and CD19 DN CAR T-cells are shown to recognize CD19 antigen on target antigen-bearing Nalm-6 cells and Z-138 cells. Non-CAR T-cells were a baseline/negative degranulation control. The CAR T-cells were gated on CD8+ EGFR+, whereas non-CAR T-cells were gated on CD8+ alone (representative data included with total repeats of $n = 3$). (B) An ELISA was used to measure granzyme B release (pg/mL) by the all the CD19-targeting CAR T-cells, in response to Nalm-6 and Z-138 target cell lines. The non-CAR T-cells were kept as a negative control (representative data shown for total repeats $n = 3$). Bar graphs plotted the means with SEM. ****$p < 0.0001$; ns, not significant. (C) The direct killing assay showed percent cytotoxicity of CD19-targeting CAR T-cells against Nalm-6-GFP cell model. The non-CAR T-cells and the CD19 KO Nalm-6 target cells served as negative controls (representative data). Refer to Figure S5 for the BAFF-R-targeted evaluation and Figure S6 for the CD107a data gated on CD4.

similar to unedited CAR T-cells (Figure 4C). This method to manufacture allogeneic CAR T-cells was performed four times with the means of our quality control (QC) parameters as summarized (Figure S4A). The CAR construct included a truncated EGFR, which doubled as a suicide switch and a CAR T-cell content/potency marker for QC metrics.[24–26] There were neither appreciable differences between the T-cell memory subsets and naive phenotype nor changes in T-cell exhaustion markers of CRAFT- and DN CAR T-cells (Figures S4B and S4C). This suggests that the gene editing protocol does not affect T-cell memory and exhaustion profiles. Based on these results, we report an efficient and streamlined protocol for the generation of enriched allogeneic CAR T-cells, where gene editing and subsequent sorting of CAR T-cells did not impede the manufacturing quality of CAR T-cells.

CRAFT CAR T-cells show potent, *in vitro* antigen-specific cytotoxicity

With quality CAR T-cells in hand, we evaluated the antigen-specific CAR T-cell function of the DN CAR T-cells and the CRAFT CAR T-cells. Nalm-6 WT cells, a CD19-positive acute lymphocytic leukemia cell line, and a CD19-KO Nalm-6 cell line were used as target cells to evaluate antigen-specific cytotoxicity of the CD19 CAR T-cells. To further validate antigen-directed response of CRAFT CAR T-cells, we used Z-138 WT cells, a mantle cell lymphoma cell line, and its derived CD19 KO version. The allogeneic CD19 CRAFT CAR T-cells and CD19 DN CAR T-cells elicited a potent antigen-specific degranulation response in both CD8 CAR T (Figure 5A) and CD4 CAR T populations (Figure S6A), as evidenced by a CD107a expression. The degranulation response was similar to CD19 unedited CAR T-cells, where non-CAR T-cells defined a baseline degranulation signal. Additionally, both genetically engineered CD19 allogeneic CAR

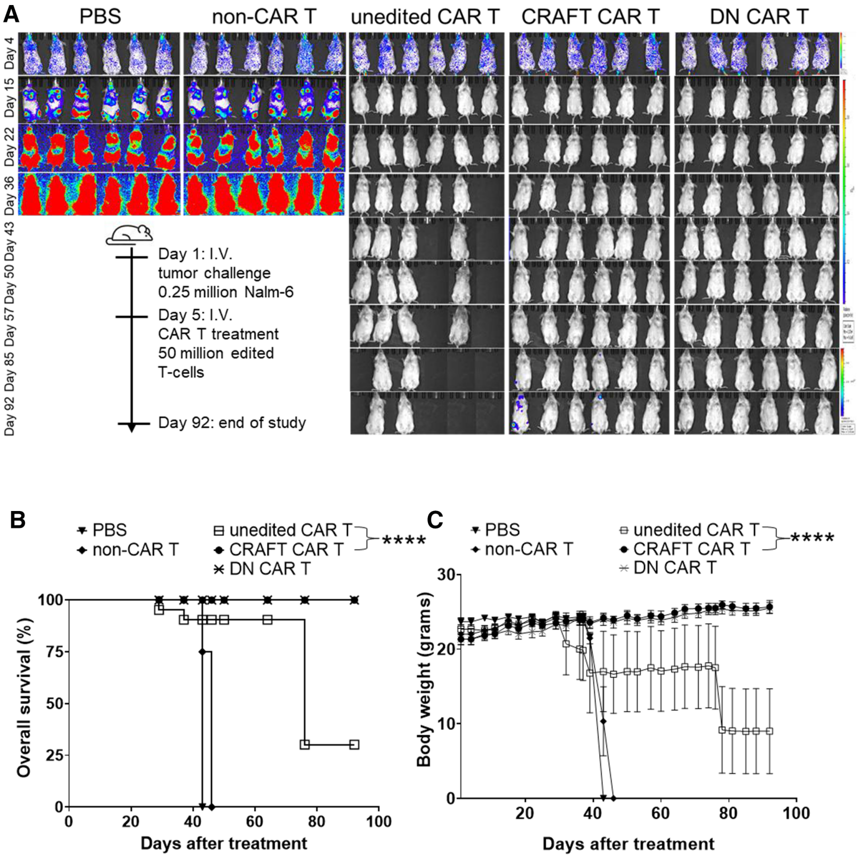

Figure 6. CRAFT CAR T-cells elicited *in vivo* anti-tumor effects without causing GVHD

(A) BLI images of individual animals following reduction of Nalm-6 leukemia burden using BAFF-R CAR T-cell treatments (5 cohorts, $n = 6$ animals per cohort). On day 1, NSG mice received 0.25 million WT Nalm-6 GFP-luciferase cells via i.v. injection, where luminescence signal indicated leukemia burden (p/sec/cm^2/sr). Animal randomization on day 4 was followed by a single dose of i.v. treatment using 50 million edited T-cells on day 5. (B) The animals were monitored for 92 days as depicted in percent survival curve. Representative Kaplan-Meier plot of overall survival data. Log rank test: $****p < 0.0001$ between unedited and CRAFT CAR T-cell cohorts. (C) The plot of mean body weight (grams) after CAR T treatment records weight loss in different treatment cohorts either due to GVHD or tumor-related cachexia. *xy* graph plotted the average with SEM. $****p < 0.0001$; ns, not significant.

T-cells released granzyme B in response to antigen stimulation by Nalm-6 and Z-138 target cells, (Figure 5B) similar to CD19 unedited CAR T-cells. CD19 allogeneic CAR T-cells also demonstrated antigen-specific cytotoxicity in a direct killing assay, in which GFP-positive engineered Nalm-6 cells were the target-bearing cells (Figure 5C). Together, these compelling results establish both allogeneic CAR T-cell fractions to be equipotent in target recognition and subsequent killing to unedited CD19 CAR T-cells.

Using BAFF-R CAR-encoding lentivirus, we manufactured unedited and allogeneic CAR T-cells and evaluated their antigen-specific cytotoxicity against BAFF-R-positive Nalm-6 cells.[27] Predictably, we observed similar rate of antigen-specific degranulation in all BAFF-R-expressing CAR T-cells in CD8 (Figure S5A) and CD4 (Figure S6B) T-cell populations. As evidenced in Figure S5B, granzyme B was released when the allogeneic BAFF-R CAR T-cells recognized antigen on the target cells. The antigen recognition by unedited, CRAFT-, and DN CAR T-cells were similar as evidenced by the directed killing of antigen-bearing WT Nalm-6 cells and the sparing of BAFF-R KO Nalm-6 control (Figure S5C). Non-CAR-T-cells showed no cytotoxicity, confirming that CAR T-cells activated and responded via CAR-intrinsic CD3ζ and costimulatory signaling domains, rather than through endogenous TCR. Overall, these results indicated that CRAFT CAR T-cells are amenable to transduction via lentivirus and that the editing does not hamper the antigen-specific response granted through the CAR. Together DN- as well as CRAFT CAR T-cells could be used to generate allogeneic CAR T-cells.

CRAFT CAR T-cells elicited *in vivo* anti-tumor effects without causing GVHD

We used a xenograft mouse model, in which mice were challenged with Nalm-6 cells, to evaluate both the *in vivo* anti-tumor effects and safety of BAFF-R CRAFT-CAR T-cells (Figure 6A). The PBS and non-CAR T-cell treatment cohorts were control groups that showed rapid progression of leukemia.[25] These animals had leukemia burden-related weight loss and were moribund by day 42. The BAFF-R unedited CAR T-cells showed potent anti-tumor effects, but significant morbidity (Figure 6B) was also observed in these tumor-free animals owing to GVHD. The loss of weight, along with severe alopecia, in four of six animals belonging to unedited CAR T cohort, were indicative of TCR alloreactivity (Figure 6C). With the complete absence of TCR, BAFF-R DN CAR T-cells served as the negative GVHD control, with no alloreactivity-related weight loss, alopecia, or related death observed. Likewise, none of the mice in the BAFF-R CRAFT CAR T-cell treatment cohort exhibited weight loss or GVHD-related symptoms. Both DN and CRAFT CAR T-cell treatments resulted in significant tumor suppression. Although tumor relapse was observed in two of six mice in CRAFT CAR T cohort at the end stage of the study, the overall survival remained comparable to that of the DN CAR T cohort. These results support the therapeutic efficacy and safety of CRAFT CAR T-cells, demonstrating that they are not alloreactive and do not induce GVHD.

CRAFT CAR T-cells can act as the effector cells for bispecific T-cell engager

The presence of surface-intact CD3 on CRAFT CAR T-cells distinguishes them from traditional DN CAR T-cells. We hypothesized

that the retention of CD3 may provide an advantage, enabling CRAFT CAR-T-cells to function as effector cells for bispecific T-cell engager (BiTE) and engage in CD3-dependent antitumor cytotoxicity. To evaluate this, we co-cultured BAFF-R CRAFT CAR T-cells with BAFF-R KO Nalm-6 cells in the presence of blinatumomab, a CD19 BiTE (CD3/CD19 bispecific antibody). The use of BAFF-R KO Nalm-6 cells was intended to eliminate the effects of antigen-specific CAR killing. BAFF-R unedited CAR T-cells and BAFF-R DN CAR T-cells were included as controls. Since CRAFT CAR T-cells and unedited CAR T-cells have intact surface CD3, blinatumomab can bridge these CAR T-cells to BAFF-R KO Nalm-6 cells, resulting in BAFF-R-independent cytotoxicity. As shown in the direct killing assays (Figures 7A and 7B) CRAFT CAR T-cells, like unedited CAR T-cells, exhibited cytotoxicity against BAFF-R KO Nalm-6 cells only in the presence of blinatumomab, which captured target tumor cells via the CD19 antigen. In contrast, with lack of cell surface CD3, DN CAR T-cells failed to induce cytotoxicity due to their inability to be engaged by blinatumomab. These findings highlight the potential of using BiTE as a consolidation treatment flowing allogeneic CRAFT CAR T-cell therapy, which may improve the persistence and overall therapeutic efficacy of CAR T-cell therapy.

DISCUSSION

Harnessing CRISPR gene editing, we developed a T-cell engineering platform that, for the first time, parses TCR activation from CD3 signaling. Using a precise AsCas12a Ultra enzyme and a novel crRNA (CRAFT crRNA) targeting the TCR β chain, we knocked out TRBC to favor MMEJ repair. This process generated the expected TCR− CD3− (DN) T-cells but also produced a novel TCR− CD3+ population that we have termed CRAFT-cells. These could be enriched and expanded and exhibited no alloreactivity *in vitro* or GVHD *in vivo*, mirroring DN T-cell attributes.

CRAFT-cells demonstrated favorable gene editing characteristics, with a predictable repair profile and minimal off-target effects. *Ex vivo*, CRAFT-CAR T-cells exhibited significantly high fold expansion compared to DN CAR T-cells in response to anti-CD3 stimulus. Both CD19 and BAFF-R CRAFT-CAR T-cells demonstrated antigen-specific cytotoxicity on par with conventional CAR T-cells, suggesting that CRAFT-cells are viable resources for allogeneic CAR T-cell therapy, possibly in combination with DN T-cells.

Notably, this is the first report using MMEJ intentionally to generate allogeneic CAR T-cells. Our CRAFT crRNA demonstrated fewer off-target effects than even those observed with MMEJ-powered DN crRNA (Figure S2E), underscoring the critical role of guide RNA design. We also confirmed that CRAFT-cells emerged specifically through site-specific TRBC abrogation, not TRAC editing.

Given our background in autologous CAR T-cell development for hematologic and solid tumors, we are well aware of the variability in patient-derived T-cell quality.[28,29] This work marks our initial step into the allogeneic space, motivated by the potential for healthy donor-derived T-cells to bypass limitations of autologous manufacturing and reduce toxicity risks like GVHD. Our CRAFT crRNA enabled the discovery of a CD3+, TCR− population, which may serve as "off-the-shelf" starting material for CAR T-cell therapies.

A previous *in silico* prediction study relied on modifying conserved regions of Trac to reduce TCR-pMHC binding.[30] Unlike previously, we uncovered naturally existing TCR−, CD3+ cells, in an elegant way, without inserting any artificial TCRs. We designed a CRISPR-based strategy to explore TRAC and TRBC regions for breakpoints conducive to CRAFT-cell generation, comparing AsCas12a Ultra and SpCas9. AsCas12a outperformed SpCas9 in editing efficiency, indel profile, MMEJ repair, and off-target minimization, hence making it preferable for therapeutic applications.

To understand how CD3 surface expression persisted despite TCR loss, we modeled top in-frame indels using AlphaFold2, an AI-powered protein structure elucidation tool. Structural prediction and consequent alignment against WT showed that in CRAFT-cells, truncated TCR β chains still preserved the spatial orientations of key domains like connecting peptide (CP), TM, and the cytoplasmic tail, thus retaining critical non-covalent contacts with CD3γ and ε (Figures 7C and 7D).[31] In contrast, DN crRNA editing disrupted the α-coiled coil region (E148) inside CP, which is critical for TCR αβ pairing, and the resultant shortening also led to a significant structural deviation from WT. These findings suggest that precise TRBC editing can silence TCR signaling while maintaining CD3 assembly.

As CAR T-cell technologies evolve, refinement focuses on engineering functional advantages, like enhanced persistence, evading rejection, and resistance to exhaustion via multiplex editing.[32,33] MMEJ-friendly AsCas12a-based platform can support such edits, enabling high-precision, low-risk modifications that could eventually allow for non-viral CAR integration at *TRBC* loci, mitigating random viral insertion risks.[34] A recent study indicates *in silico* off-target predicting tools to be more inclusive at off-target discovery than empirical sequencing methods.[35] Herein, we have used multiple *in silico* tools to comprehensively report off-target predictions. Currently, we are also performing additional unbiased sequencing to query the entire genome after MMEJ-favored multiplexed gene editing on AsCas12a Ultra platform. Beyond generating functional KO, this crRNA platform demonstrates that protein function can be modulated without dismantling entire complexes. Such precision opens avenues for protein interface replacement strategies through gene editing techniques. The same CRISPR strategy may also be adapted for editing induced pluripotent stem cells to develop universal cell banks with desired edits.

Our allogeneic CRAFT-CAR T-cells demonstrate efficacy and initial safety, supporting their advancement toward clinical testing. AsCas12a Ultra, with its superior specificity and multiplexing capacity, is especially suited for building armored T-cell therapies resistant to

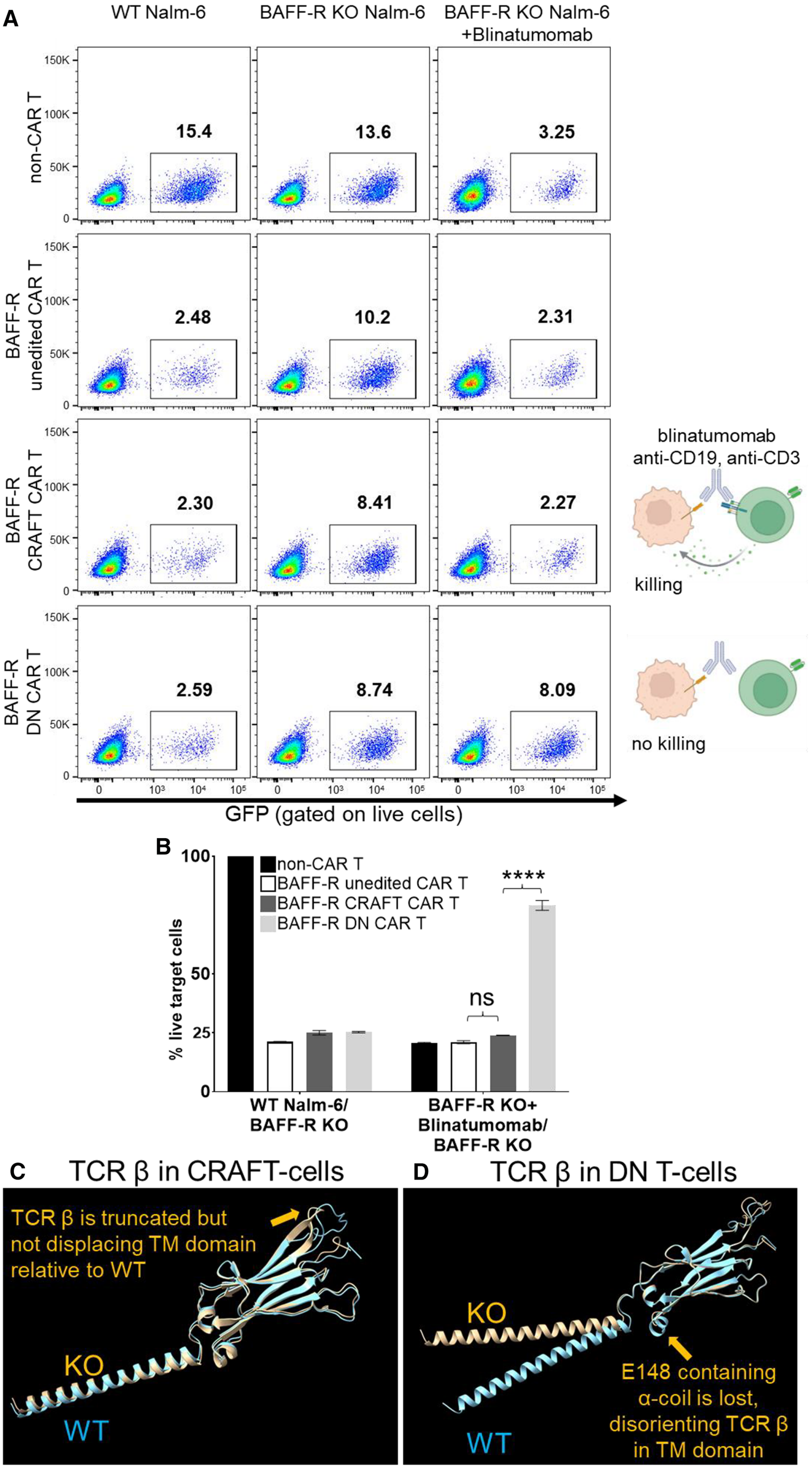

Figure 7. CRAFT CAR T-cells can act as the effector cells for BiTE

(A) Representative data showing percent cytotoxicity of Nalm-6-GFP cells when co-cultured with various BAFF-R-targeting CAR T-cells. BAFF-R KO Nalm-6 target cells served as negative controls and could only be targeted when blinatumomab (30 ng/mL) was added to the co-culture (n = 2 different donors). CAR T illustration highlighting the killing pathway was created with BioRender.com. (B) Cytotoxicity data are plotted to show percent killing of antigen-negative, BAFF-R KO Nalm-6 cells by T-cells, when blinatumomab was included. This is indicated by percent of (BAFF-R KO target cells+ blinatumomab/BAFF-R KO target cells). Percent of (WT Nalm-6 target cells/BAFF-R KO target cells) in co-culture served as positive control. Bar graphs plotted the means with SEM. ****$p < 0.0001$; ns, not significant. (C and D) Comparison of putative protein structures for TCR β in CRAFT-cells and TCR β in DN T-cells. TRBC1 protein structure (NCBI: GenBank: AAC80213.1) was imported into AlphaFold2 software to generate the ribbon protein structure of the WT TCR β subunit (blue). Putative ribbon structures (orange) for edited TCR β chains in T-cells edited with the (C) CRAFT crRNA or (D) the DN crRNA using AlphaFold2 (Google DeepMind) were predicted. Proteins containing indel sequence #3 and indel sequence #1 (in-frame) afforded after CRAFT crRNA or DN crRNA editing, respectively, were used for predicting structures. Using Needleman-Wunsch algorithm, the Ig-like domains are aligned (using ChimeraX: https://www.rbvi.ucsf.edu/chimerax) to show the putative global conformational changes that resulted from gene editing. The TCR β subunit in the CRAFT-cell shows a modest change from the WT that is concentrated to the loop region in the extracellular Ig-like domain (highlighted by the arrow). The putative structural changes to the TCR β subunit in the DN T-cells is more significant with a loss of an alpha helix that introduces a significant bend to TCR β subunit that potentially disrupts the formation of the octameric TCR complex. Arrows show the KO region.

rejection and exhaustion.[36–39] We are gearing up to translate our CAR T strategy into a cGMP-compliant protocol, which will be using GMP-grade enzymes to produce a fully allogeneic multi-edited, CAR T product, devoid of GVHD or HVG interactions. As we previously described, using T-cells enriched in naive, stem cell-like, and central memory profile (Tn/mem) for CAR T manufacture improves immunotherapeutic persistance.[40] Since no precise engineering, donor exhaustion profiling, or use of Tn/mem as starting T-cell material were tested to alleviate exhaustion specifically, T-cell exhaustion was not systematically evaluated in this manuscript. Future studies should specifically address how exhaustion may affect these cells to evaluate long-term functionality, especially after multi-antigen exposure.

In the animal tumor challenge study, 67% of CRAFT CAR T-treated animals remained tumor free, as compared to 33% overall survival in unedited CAR T-cell cohort. The recurring tumor burden in two of six animals in CRAFT CAR T cohort could probably be due to (among other reasons) the increased leukemia burden outnumbering CAR T-cells or the inability of CAR T-cells to form meaningful memory inside a xenophobic environment devoid of any human cytokine support.

Importantly, CRAFT-cells maintained CD3 expression and were detectable *in vivo* for up to 2 weeks longer than DN T-cells. This makes them promising candidates for combinatorial strategies, particularly with CD3-targeting bispecific antibodies (e.g., BiTEs) like blinatumomab, or BAFF-R BiTE.[41–43] We demonstrate the selective CD3-mediated tumor ablative response of CRAFT-CAR T-cells as functional effector cells of BiTE. This uniqueness distinguishes CRAFT from DN T-cells, while widening the clinical applications to a consolidated immunotherapeutic approach to treat minimal residual disease or indolent lymphomas.[44]

Since the overall yield of CRAFT-CAR T is 10%, a dual-infusion strategy, delivering both DN T- and CRAFT-cells in an optimized ratio, may offer an immediate anti-tumor effect via DN T-cells and sustained activity through CD3+ CRAFT-cells activated by BiTE antibodies. This platform could help improve CAR T durability by enabling repeated T-cell engagement and prolonging tumor control in patients. These findings lay the groundwork for incorporating CRAFT-cells in designing more durable next-generation allogeneic immunotherapies.

MATERIALS AND METHODS

A detailed materials list is included in Table S1.

Cell lines and culturing conditions

Nalm-6 and Z-138 cells were purchased from ATCC, where Nalm-6 were cultured using RPMI 1640 medium and Z-138 cells were maintained using IMDM media each supplemented with 10% fetal bovine serum. As previously described, the cells lines were first engineered to overexpress luciferase-GFP, clonally selected, and expanded from a single cell to be termed as “WT” in this study. WT-Nalm-6 cells were then edited to either KO CD19 or BAFF-R expression, whereas WT-Z-138 cells were edited to KO CD19.[25] KO cell lines were established from single-cell clone after cell sorting. The antigen-specific expression of target cell lines was authenticated via flow cytometry before cryopreservation. All cell clones were assessed periodically for mycoplasma contamination and authenticated via ATCC.

Pan T-cell isolation from healthy donor blood samples

As described previously, healthy donor-derived peripheral blood mononuclear cells (PBMCs) were obtained via leukapheresis using leukocyte reduction system cones, by the Division of Transfusion Medicine, Mayo Clinic, Rochester, Minnesota, following current regulatory requirements.[45] The contents of leukapheresis cone were diluted in PBS, and the isolation of mononuclear cells was performed using Ficoll gradient centrifugation, following established methods.[25] Pan T-cells were isolated following the kit instructions to collect the negative fraction from LS columns. The positive fractions extruded from pan T-cell isolation kit were cryopreserved to generate the mature DCs necessary for other experiments. T-cells were cultured in a complete T-cell culture media composed of X-VIVO 15 base media, supplemented with 10% human serum, with final concentration of 50 U/mL recombinant human IL2 (rh-IL2) and 0.5 ng/mL recombinant human IL15 (rh-IL15).

CRISPR-Cas-based gene editing

To generate CRAFT-cells, both AsCas12a-compatible crRNA and SpCas9-compatible sgRNA were designed to target a gene sequence located within a consensus sequence that is common to exon 1 of both TCR β constant regions 1 and 2. To generate DN T-cells, AsCas12a-compatible crRNA was used, which also targeted another consensus sequence commonly shared between exon 1 of TRBC1 and 2. The sequences used to target *TCR β* in this study were following: CRAFT crRNA: GCCCTATCCTGGGTCCACTCG; TRBC sgRNA: GGAGAATGACGAGTGGACCC; and DN TRBC crRNA: GGTGTGGGAGATCTCTGCTTC. CRAFT and DN crRNAs were selected bearing MMEJ sites nearby. For AsCas12a Ultra-assisted TCR α (TRAC) KO, the sequence used was TRAC crRNA: CACATGCAAAGTCAGATTTGT. The RNP complexation between the enzyme and gRNAs followed the instructions available on product website. Four days after anti-CD3/CD28 bead stimulation, primary human T-cells were de-beaded, counted, washed, and resuspended in Lonza nucleofection buffer P3. Enzyme-compatible electroporation enhancer and matching RNP complex were electroporated into the T-cells by applying Lonza nucleofector 4D program EH155. Both enzymes used were research-grade (IDT) specially designed to significantly reduce off-targeting.

CAR T-cell generation

Second-generation, self-inactivating lentiviral CD19 and BAFF-R CAR constructs with the configurations described previously were used.[25] Pan T-cells were activated using anti-CD3/CD28 beads overnight before transfection with CD19 or BAFF-R CAR packaged lentivirus. After transfection, the CAR T-cells were

cultured for 72 h; these CAR T-cells were subjected to gene editing to produce the desired CRAFT-cells or DN CAR T-cells. After 72 h, the TCR+ fraction from edited CAR T-cells was depleted following the instructions of TCR a/b depletion kit and sorted to enrich CRAFT CAR T- and DN CAR T-cells. CAR T-cells were expanded for 7–9 days in complete T-cell culture media.

Analysis of gene editing signatures via sequencing

DNA was extracted from frozen cell pellets (10×10^6 cells each) using a QIAamp DNA mini kit. Target regions of interest were amplified via designing fusion primers that contained proprietary MiSeq adapter sequences fused to target sequences, and unique barcode sequences were then added to each amplicon. A pooled DNA library was sequenced on MiSeq flow cell as a 2×150 bp, paired-end sequencing run. Demultiplexed datasets were subjected to read cleanup protocol of adapter trimming before analysis and alignment against corresponding reference sequences. Short read-sequences were discarded before assembling on-target read pairs, and then a Needleman-Wunsch-based alignment of sequences was performed against WT. Deletions and insertions were quantified as characteristic events of NHEJ, and their frequency was calculated for each target site (Cas-Analyzer). The 5′-3′ fusion primers used to enquire were as follows: TRBC1 & 2: GGTCTCGGCCACCTTCTGGCA GAACCCCCGCAACCACTTCCGCTGTCAAGTCCAGTTCTACG GGCTCTCGGAGAATGACGAGTGGACCCAGGATAGGGCCAA ACCCGTCACCCAGATCGTCAGCGCCGAGGCCTGGGGTAGAG CAGGTGAGTGGGGCCTGGGGAGATGCCTGGAGGAGATTAGG, and **TRAC**: CTGCTCTGGATGCTGAAAGAATGTCTGTTTTTCCT TTTAGAAAGTTCCTGTGATGTCAAGCTGGTCGAGAAAAGCTT TGAAACAGGTAAGACAGGGGTCTAGCCTGGGTTTGCACAG GATTGCGGAAGTGATGAACCCGCAATAACCCTGCCTGGAT GAGGGAGTGGGAAGAAATT.

The off-target analysis was performed using CCTop program and corroborated using Cas-OFFinder, using a mismatch value of 4. Being Cas9 specific, COSMID analysis was excluded from *in silico* analysis. Even though CRISPOR yielded no exonic off-targets using crRNA+ AsCas12a, the tool was punitive against SpCas9; therefore, analysis was not included.

PBMC-derived allogeneic dendritic cells and *in vitro* alloreactivity assay

PBMCs from multiple healthy donors were pooled and used for DC maturation. DCs were generated using published protocols with minor modifications.[4,46] DCs were cultured in RPMI-1640 medium with 10% human serum, 1% penicillin/streptomycin, and 2% glutamine. PBMCs were first activated with cytokine support of granulocyte-macrophage colony-stimulating factor (GM-CSF) (800 U/mL) and interleukin (IL)-4 (500 U/mL) on day 1 and then left to culture over a 6-day period. On day 6 in addition to GM-CSF (800 U/mL) and IL-4 (500 U/mL), DC polarization was induced by supplementation of TNF-α (10 ng/mL), IL-1β (13.2 ng/mL), IL-6 (10 ng/mL), and PGE_2 (1 μg/mL) and DCs were allowed to grow for an additional 48–72 h. Following DC polarization, the cells were harvested, counted, and their phenotype expression was confirmed via flow cytometry (Figure S2D). Multi-donor-derived, allo-DCs were plated and subsequently irradiated with 40 Gy (stimulator), to reduce any confounding factors e.g., host-versus-graft interaction. Edited T-cells (responder) were then plated, where the co-cultured cells were in DC:T-cell ratio of 1:10. The co-culture was incubated for at 96–120 h[47] T-cells and DCs were also plated alone as controls. The cells were collected and stained using EdU incorporation assay following the manufacturer's protocol (Thermo Fisher) and validated via flow cytometry.

Flow cytometry

The phenotype of edited T-cells was verified using BV605-CD3 and APC-TCRα/β antibodies via BD LSRII Fortessa cell analyzer. The antibody panel used to discern DC maturation included staining for BV650-CD14, PE-HLADR, BV421-CD11c, APC-CD209, BV786-CCR7, and PerCP-Cy5.5A-CD80. All events were gated on live cells before acquisition, and data were analyzed using FlowJo v.10 software.

In vivo studies

Animal GVHD studies

NSG mouse breeding pairs were purchased from The Jackson Laboratory (stock no. 005557) to establish a breeding colony that was monitored in a pathogen-free animal facility at the Animal Resource Center at Mayo Clinic Florida, per institutional guidelines. Animal studies were approved by and in accordance with guidelines of the Institutional Animal Care and Use Committee (IACUC: 15020; protocol number A00005759). For achieving transient immune suppression to accelerate the onset and progression of xenogeneic GVHD, mice (6–8 weeks) were first exposed to total-body irradiation of sublethal X-ray dose (2 Gy). A day later, animals were randomized to four separate groups (n = 4 or 5 per repeat study) and then received an intravenous (i.v.) treatment injection containing one of four treatments: unedited T-cells, CRAFT-cells, DN T-cells, or PBS. Treatment consisted of 50×10^6 of the test T-cells resuspended in ice-cold, sterile PBS. The body weights were measured once every 7 days for the duration of 107 days. Animals were evaluated for signs of GVHD twice per week by body condition scoring that included hunching, alopecia, reduced activity, and changes in shape of nose. Mice were humanely euthanized if 15% body weight was lost over a period of 3 consecutive days or 20% of body weight loss after the day of irradiation.[4] During the study, mice were anesthetized, and blood was collected via submandibular bleed and stored in heparin tubes. Blood red blood cells were lysed using ACK lysis buffer for 20 min and washed, and the resulting cells were stained to evaluate via flow cytometry to confirm human T-cell engraftment. The antibody panel included PE-CD45, BV605-CD3, APC-TCR, and Sytox blue.

In vivo *anti-tumor studies*

NSG mice aged 6–10 weeks were injected with 0.25×10^6 cells i.v. on day 1. On day 4, the animals were imaged using Revvity IVIS instrument and followed by randomization. In addition to evaluating

anti-tumor effects, to assess alloreactivity, a bolus shot of edited T-cell treatment (50×10^6 cells) was administered i.v. on day 5. The leukemia burden was monitored weekly; the animals were periodically weighed (twice a week) and evaluated for signs of GVHD and/or tumor-related morbidity.

In vitro functional assays

All *in vitro* assay methodology has been reported previously; discussed below are specific details that pertain to the current work.[25,26]

Degranulation assay

Effector T-cells were co-incubated for 6 h with target cells at an effector-to-target (E:T) ratio of 2:1 resuspended in 10% FBS-supplemented RPMI 1640 medium containing no cytokines. To visualize granule membrane localized on T-cell surface, GolgiStop Protein Transport Inhibitor Reagent and CD107a APC antibody were added during the incubation period. The cells were subsequently stained with antibodies against BV605-CD3, PE-Cy7-CD4, APC-Cy7-CD8, and BV421-EGFR. Samples were acquired using BD LSRII Fortessa flow cytometer.

Granzyme B ELISA assay

T-cells and target cells were co-cultured for 72 h at E:T ratio of 4:1 in cytokine-free complete media. Cell culture supernates were carefully collected, and granzyme B protein release was quantified using human granzyme B assay kit following manufacturer's instructions.

Direct killing assay

T-cells and GFP labeled-target cells were co-incubated for 24 h at E:T of 20:1, in cytokine-free complete media. The cells were subsequently washed and stained with live/dead marker before acquiring data on Fortessa. Each experiment was conducted in triplicate. The CAR T-cell-induced direct killing is represented by loss in GFP expression relative to the CAR T-cell co-culture with antigen-negative target cells. Blinatumomab studies were conducted at E:T ratio of 4:1 for 12 h, with a final concentration of 30 ng/mL.

Statistical analysis

Statistical analyses were conducted using GraphPad Prism 7. Paired, parametric, two-tailed Student t test was used to compute the statistical significance between experimental conditions of at least three separate experiments. Mann-Whitney U test was applied to calculate statistical significance between animal weights in different cohorts. Overall survival of mice was analyzed using Kaplan-Meier curve. Tukey's multiple comparison test was used to evaluate significance between two groups that compared multiple time points. Statistical significance has been indicated as follows, ****$p < 0.0001$, ***$p < 0.001$, **$p < 0.01$, *$p < 0.05$; not significant (ns) if $p > 0.05$.

DATA AVAILABILITY

Data were generated by the authors and are included in the article.

ACKNOWLEDGMENTS

We would like to acknowledge the funding support to H.Q. inluding the Florida Health Grant (#MOG07), the Mayo Clinic Florida CAR-T Manufacturing Program fund and Casey DeSantis Cancer Research fund. We are grateful to Dr. Stephen Ekker for his invaluable input on available MMEJ resources. We recognize Mayo Clinic-Florida's Histology Core and Cytometry and Cell Imaging Core (CCIC) for their support and resource sharing.

AUTHOR CONTRIBUTIONS

H.Q., T.H., and M.A.K.-D. created the study concept and designed the studies. T.H., Y.L., Y.Q., A.D.B., I.V.-R., and S.G. performed experiments and conducted data analysis. T.H. and M.E.G. prepared figures. T.H., M.E.G., Y.L., S.G., and H.Q. collaborated in the writing and editing of the manuscript.

DECLARATION OF INTERESTS

The authors declare no competing interests.

DECLARATION OF GENERATIVE AI AND AI-ASSISTED TECHNOLOGIES IN THE WRITING PROCESS

During the preparation of this work the authors used ChatGPT to limit section word count. After using tool, the authors reviewed and edited the content as needed and take full responsibility for the content of the publication.

SUPPLEMENTAL INFORMATION

Supplemental information can be found online at https://doi.org/10.1016/j.omtn.2025.102778.

REFERENCES

1. Klaver, Y., van Steenbergen, S.C.L., Sleijfer, S., Debets, R., and Lamers, C.H.J. (2016). T Cell Maturation Stage Prior to and During GMP Processing Informs on CAR T Cell Expansion in Patients. Front. Immunol. *7*, 648.
2. Cranert, S.A., Richter, M., Tong, M., Weiss, L., Tan, Y., Ostertag, E.M., Coronella, J., and Shedlock, D.J. (2019). Manufacture of an Allogeneic CAR-T Stem Cell Memory Product Candidate for Multiple Myeloma, P-Bcma-ALLO1, Is Robust, Reproducible and Highly Scalable. Blood *134*, 4445.
3. Qasim, W., Zhan, H., Samarasinghe, S., Adams, S., Amrolia, P., Stafford, S., Butler, K., Rivat, C., Wright, G., Somana, K., et al. (2017). Molecular remission of infant B-ALL after infusion of universal TALEN gene-edited CAR T cells. Sci. Transl. Med. *9*, eaaj2013.
4. Ren, J., Liu, X., Fang, C., Jiang, S., June, C.H., and Zhao, Y. (2017). Multiplex Genome Editing to Generate Universal CAR T Cells Resistant to PD1 Inhibition. Clin. Cancer Res. *23*, 2255–2266.
5. Call, M.E., Pyrdol, J., Wiedmann, M., and Wucherpfennig, K.W. (2002). The organizing principle in the formation of the T cell receptor-CD3 complex. Cell *111*, 967–979.
6. Krshnan, L., Park, S., Im, W., Call, M.J., and Call, M.E. (2016). A conserved alpha-beta transmembrane interface forms the core of a compact T-cell receptor-CD3 structure within the membrane. Proc. Natl. Acad. Sci. USA *113*, E6649–E6658.
7. Lonez, C., and Breman, E. (2024). Allogeneic CAR-T Therapy Technologies: Has the Promise Been Met? Cells *13*, 146.
8. Kocoglu, M.H., Asch, A., Ramakrishnan, A., Bachier, C., Martin, T., Rodriguez, T., McArthur, K., Martin, C., Namini, H., Ostertag, E., et al. (2022). 47P Phase I study to assess the safety and efficacy of P-BCMA-ALLO1: A fully allogeneic CAR-T therapy, in patients with relapsed/refractory multiple myeloma (RRMM). Immun. Oncol. Technol. *16*.
9. Moradi, V., Omidkhoda, A., and Ahmadbeigi, N. (2023). The paths and challenges of "off-the-shelf" CAR-T cell therapy: An overview of clinical trials. Biomed. Pharmacother. *169*, 115888.
10. Stenger, D., Stief, T.A., Kaeuferle, T., Willier, S., Rataj, F., Schober, K., Vick, B., Lotfi, R., Wagner, B., Grünewald, T.G.P., et al. (2020). Endogenous TCR promotes in vivo persistence of CD19-CAR-T cells compared to a CRISPR/Cas9-mediated TCR knockout CAR. Blood *136*, 1407–1418.

11. Ata, H., Ekstrom, T.L., Martínez-Gálvez, G., Mann, C.M., Dvornikov, A.V., Schaefbauer, K.J., Ma, A.C., Dobbs, D., Clark, K.J., and Ekker, S.C. (2018). Robust activation of microhomology-mediated end joining for precision gene editing applications. PLoS Genet. *14*, e1007652.

12. Martinez-Galvez, G., Joshi, P., Friedberg, I., Manduca, A., and Ekker, S.C. (2021). Deploying MMEJ using MENdel in precision gene editing applications for gene therapy and functional genomics. Nucleic Acids Res. *49*, 67–78.

13. Hu, Y., Zhou, Y., Zhang, M., Ge, W., Li, Y., Yang, L., Wei, G., Han, L., Wang, H., Yu, S., et al. (2021). CRISPR/Cas9-Engineered Universal CD19/CD22 Dual-Targeted CAR-T Cell Therapy for Relapsed/Refractory B-cell Acute Lymphoblastic Leukemia. Clin. Cancer Res. *27*, 2764–2772.

14. Pal, S.K., Tran, B., Haanen, J.B., Hurwitz, M., Sacher, A., Agarwal, N., Tannir, N., Budde, E., Harrison, S., Klobuch, S., et al. (2024). Abstract CT002: CTX130 allogeneic CRISPR-Cas9-engineered chimeric antigen receptor (CAR) T cells in patients with advanced clear cell renal cell carcinoma: Long-term follow-up and translational data from the phase 1 COBALT-RCC study. Cancer Res. *84*, CT002.

15. Wei, W., Chen, Z.N., and Wang, K. (2023). CRISPR/Cas9: A Powerful Strategy to Improve CAR-T Cell Persistence. Int. J. Mol. Sci. *24*, 12317.

16. Stadtmauer, E.A., Fraietta, J.A., Davis, M.M., Cohen, A.D., Weber, K.L., Lancaster, E., Mangan, P.A., Kulikovskaya, I., Gupta, M., Chen, F., et al. (2020). CRISPR-engineered T cells in patients with refractory cancer. Science *367*, eaba7365.

17. Haapaniemi, E., Botla, S., Persson, J., Schmierer, B., and Taipale, J. (2018). CRISPR-Cas9 genome editing induces a p53-mediated DNA damage response. Nat. Med. *24*, 927–930.

18. Wen, W., Quan, Z.J., Li, S.A., Yang, Z.X., Fu, Y.W., Zhang, F., Li, G.H., Zhao, M., Yin, M.D., Xu, J., et al. (2021). Effective control of large deletions after double-strand breaks by homology-directed repair and dsODN insertion. Genome Biol. *22*, 236.

19. Zhang, L., Zuris, J.A., Viswanathan, R., Edelstein, J.N., Turk, R., Thommandru, B., Rube, H.T., Glenn, S.E., Collingwood, M.A., Bode, N.M., et al. (2021). AsCas12a ultra nuclease facilitates the rapid generation of therapeutic cell medicines. Nat. Commun. *12*, 3908.

20. Xin, C., Yin, J., Yuan, S., Ou, L., Liu, M., Zhang, W., and Hu, J. (2022). Comprehensive assessment of miniature CRISPR-Cas12f nucleases for gene disruption. Nat. Commun. *13*, 5623.

21. He, S., Lu, Y., Liu, X., Huang, X., Keller, E.T., Qian, C.N., and Zhang, J. (2015). Wnt3a: functions and implications in cancer. Chin. J. Cancer *34*, 554–562.

22. Yang, C.W., Hojer, C.D., Zhou, M., Wu, X., Wuster, A., Lee, W.P., Yaspan, B.L., and Chan, A.C. (2016). Regulation of T Cell Receptor Signaling by DENND1B in TH2 Cells and Allergic Disease. Cell *164*, 141–155.

23. Poirot, L., Philip, B., Schiffer-Mannioui, C., Le Clerre, D., Chion-Sotinel, I., Derniame, S., Potrel, P., Bas, C., Lemaire, L., Galetto, R., et al. (2015). Multiplex Genome-Edited T-cell Manufacturing Platform for "Off-the-Shelf" Adoptive T-cell Immunotherapies. Cancer Res. *75*, 3853–3864.

24. Qin, H., Dong, Z., Wang, X., Cheng, W.A., Wen, F., Xue, W., Sun, H., Walter, M., Wei, G., Smith, D.L., et al. (2019). CAR T cells targeting BAFF-R can overcome CD19 antigen loss in B cell malignancies. Sci. Transl. Med. *11*, eaaw9414.

25. Luo, Y., Qie, Y., Gadd, M.E., Manna, A., Rivera-Valentin, R., To, T., Li, S., Yassine, F., Murthy, H.S., Dronca, R., et al. (2023). Translational development of a novel BAFF-R CAR-T therapy targeting B-cell lymphoid malignancies. Cancer Immunol. Immunother. *72*, 4031–4047.

26. Qie, Y., Gadd, M.E., Shao, Q., To, T., Liu, A., Li, S., Rivera-Valentin, R., Yassine, F., Murthy, H.S., Dronca, R., et al. (2024). Targeting chronic lymphocytic leukemia with B-cell activating factor receptor CAR T cells. MedComm *5*, e716.

27. Dong, Z., Cheng, W.A., Smith, D.L., Huang, B., Zhang, T., Chang, W.C., Wang, X., Forman, S.J., Kwak, L.W., and Qin, H. (2020). Antitumor efficacy of BAFF-R targeting CAR T cells manufactured under clinic-ready conditions. Cancer Immunol. Immunother. *69*, 2139–2145.

28. Jarmi, T., Luo, Y., Attieh, R.M., Qie, Y., Gadd, M.E., Hundal, T., Mao, S., Murthy, H.S., Taner, B.C., Kharfan-Dabaja, M.A., and Qin, H. (2024). CAR T-cell therapy-paving the way for sensitized kidney transplant patients. Kidney Int. *105*, 1124–1129.

29. Luo, Y., Gadd, M.E., Qie, Y., Otamendi-Lopez, A., Sanchez-Garavito, J.E., Brooks, M.M., Ulloa Navas, M.J., Hundal, T., Li, S., Jones, V.K., et al. (2024). Solid cancer-directed CAR T cell therapy that attacks both tumor and immunosuppressive cells via targeting PD-L1. Mol. Ther. Oncol. *32*, 200891.

30. Brazin, K.N., Mallis, R.J., Boeszoermenyi, A., Feng, Y., Yoshizawa, A., Reche, P.A., Kaur, P., Bi, K., Hussey, R.E., Duke-Cohan, J.S., et al. (2018). The T Cell Antigen Receptor alpha Transmembrane Domain Coordinates Triggering through Regulation of Bilayer Immersion and CD3 Subunit Associations. Immunity *49*, 829–841.e6.

31. Dong, D., Zheng, L., Lin, J., Zhang, B., Zhu, Y., Li, N., Xie, S., Wang, Y., Gao, N., and Huang, Z. (2019). Structural basis of assembly of the human T cell receptor-CD3 complex. Nature *573*, 546–552.

32. Lynn, R.C., Weber, E.W., Sotillo, E., Gennert, D., Xu, P., Good, Z., Anbunathan, H., Lattin, J., Jones, R., Tieu, V., et al. (2019). c-Jun overexpression in CAR T cells induces exhaustion resistance. Nature *576*, 293–300.

33. Zhang, X., Jin, X., Sun, R., Zhang, M., Lu, W., and Zhao, M. (2022). Gene knockout in cellular immunotherapy: Application and limitations. Cancer Lett. *540*, 215736.

34. Eyquem, J., Mansilla-Soto, J., Giavridis, T., van der Stegen, S.J.C., Hamieh, M., Cunanan, K.M., Odak, A., Gönen, M., and Sadelain, M. (2017). Targeting a CAR to the TRAC locus with CRISPR/Cas9 enhances tumour rejection. Nature *543*, 113–117.

35. Cromer, M.K., Majeti, K.R., Rettig, G.R., Murugan, K., Kurgan, G.L., Bode, N.M., Hampton, J.P., Vakulskas, C.A., Behlke, M.A., and Porteus, M.H. (2023). Comparative analysis of CRISPR off-target discovery tools following ex vivo editing of CD34(+) hematopoietic stem and progenitor cells. Mol. Ther. *31*, 1074–1087.

36. Torikai, H., Reik, A., Soldner, F., Warren, E.H., Yuen, C., Zhou, Y., Crossland, D.L., Huls, H., Littman, N., Zhang, Z., et al. (2013). Toward eliminating HLA class I expression to generate universal cells from allogeneic donors. Blood *122*, 1341–1349.

37. Ottaviano, G., Georgiadis, C., Gkazi, S.A., Syed, F., Zhan, H., Etuk, A., Preece, R., Chu, J., Kubat, A., Adams, S., et al. (2022). Phase 1 clinical trial of CRISPR-engineered CAR19 universal T cells for treatment of children with refractory B cell leukemia. Sci. Transl. Med. *14*, eabq3010.

38. Diorio, C., Murray, R., Naniong, M., Barrera, L., Camblin, A., Chukinas, J., Coholan, L., Edwards, A., Fuller, T., Gonzales, C., et al. (2022). Cytosine base editing enables quadruple-edited allogeneic CART cells for T-ALL. Blood *140*, 619–629.

39. Ueda, T., Shiina, S., Iriguchi, S., Terakura, S., Kawai, Y., Kabai, R., Sakamoto, S., Watanabe, A., Ohara, K., Wang, B., et al. (2023). Optimization of the proliferation and persistency of CAR T cells derived from human induced pluripotent stem cells. Nat. Biomed. Eng. *7*, 24–37.

40. Luo, Y., Qie, Y., Gadd, M.E., Hundal, T., Brim, A.D., Rosario, I.K.V., and Qin, H. (2025). A new sort of cells for chimeric antigen receptor T-cell therapies-isolating CD14(-)CD127(+) T cells for chimeric antigen receptor T-cell manufacture. Cytotherapy *27*, 980–990.

41. Qin, X., Ning, W., Liu, H., Liu, X., Luo, W., and Xia, N. (2024). Stepping forward: T-cell redirecting bispecific antibodies in cancer therapy. Acta Pharm. Sin. B *14*, 2361–2377.

42. Qin, H. (2020). BAFF - R BISPECIFIC T - CELL ENGAGER ANTIBODY. In City of Hope, DC, *vol. US20200199232A1*, https://patentimagesstoragegoogleapiscom/1d/d6/51/5db892434ab4c2/US20200199232A1pdf.

43. Kapetanovic, E., Weber, C.R., Bruand, M., Pöschl, D., Kucharczyk, J., Hirth, E., Dietsche, C., Khan, R., Wagner, B., Belli, O., et al. (2024). Engineered allogeneic T cells decoupling T-cell-receptor and CD3 signalling enhance the antitumour activity of bispecific antibodies. Nat. Biomed. Eng. *8*, 1665–1681.

44. Radhakrishnan, V.S., and Davies, A.J. (2023). Bispecific antibodies in indolent B-cell lymphomas. Front. Immunol. *14*, 1295599.

45. Dietz, A.B., Bulur, P.A., Emery, R.L., Winters, J.L., Epps, D.E., Zubair, A.C., and Vuk-Pavlović, S. (2006). A novel source of viable peripheral blood mononuclear cells from leukoreduction system chambers. Transfusion *46*, 2083–2089.

46. Rollins, M.R., Spartz, E.J., and Stromnes, I.M. (2020). T Cell Receptor Engineered Lymphocytes for Cancer Therapy. Curr. Protoc. Immunol. *129*, e97.

47. Nguyen, X.D., Eichler, H., Dugrillon, A., Piechaczek, C., Braun, M., and Klüter, H. (2003). Flow cytometric analysis of T cell proliferation in a mixed lymphocyte reaction with dendritic cells. J. Immunol. Methods *275*, 57–68.

OMTN, Volume *36*

Supplemental information

Novel allogeneic CAR T-cell platform involving microhomology-mediated end joining repair and low off-targeting potential

Tanya Hundal, Yan Luo, Yaqing Qie, Martha E. Gadd, Andrew D. Brim, Isas Vazquez-Rosario, Shaohua Guo, Mohamed A. Kharfan-Dabaja, and Hong Qin

Supplemental figures and legends

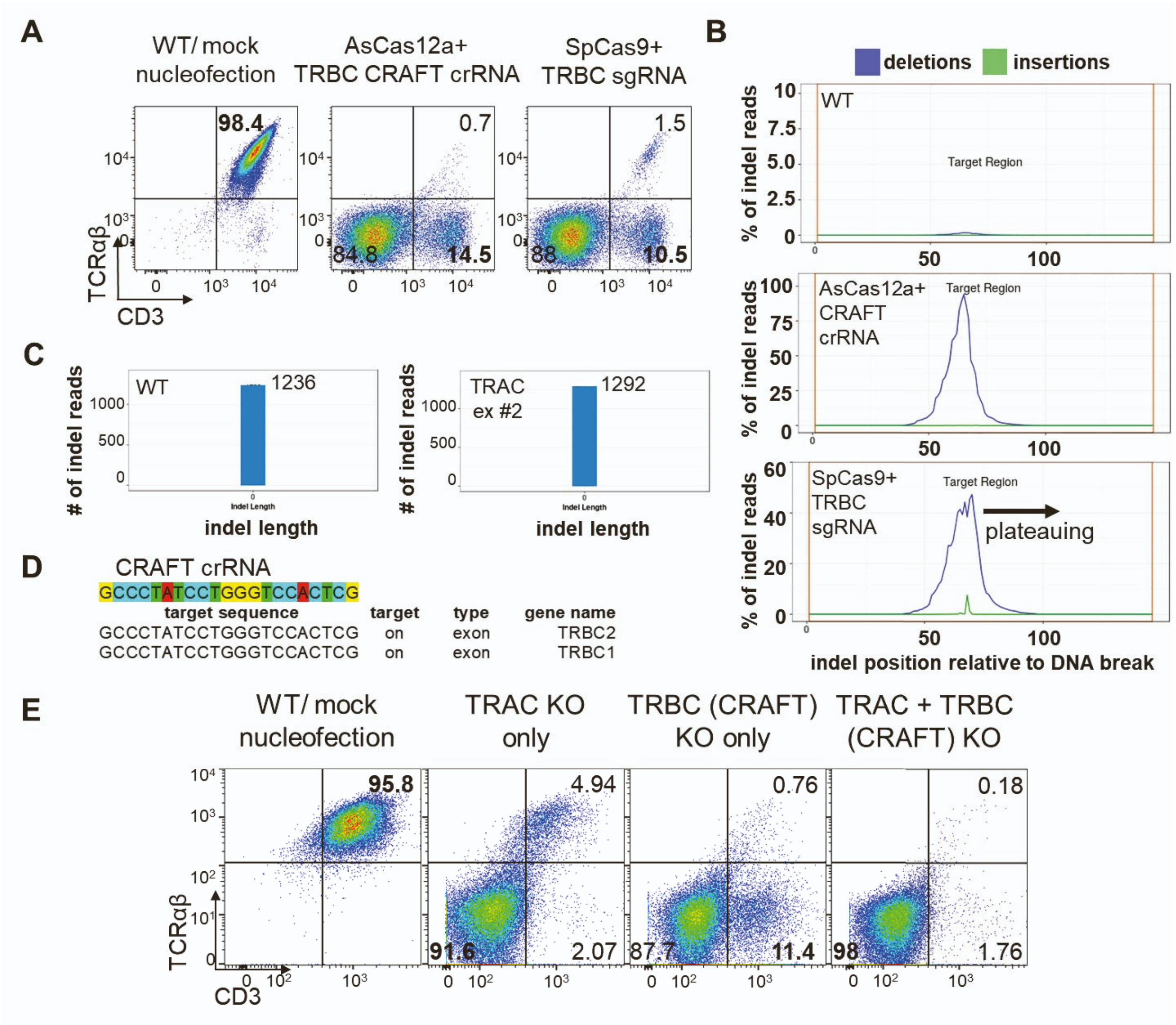

Figure S1. AsCas12a/CRAFT crRNA-assisted TCR β editing has an improved indel profile over comparable SpCas9 editing, *related to Figure 1*. A Protein expression showing generation of CRAFT-cells after TCR β KO via two strategies: AsCas12a Ultra/ TRBC crRNA (CRAFT crRNA) pair or via SpCas9/ TRBC sgRNA pair. Both pairs efficiently abrogated TCR and yielded similar percentages of TCR-,CD3+ CRAFT-cells, as determined by flow cytometry. **B** Graphs show the relative comparison of percentage of indels sequenced (Y-axis) versus the locations of indels with respect to the double stranded break (DSB) site within the target region. Zero on X-axis indicates the location of the primer probe during sequencing. The number of WT, AsCas12a, and SpCas9 reads equals 7566750, 5605620, and 6010501, respectively. **C** Bar graph comparing the number of indels generated in WT and human TRAC gene (exon 2). Indels generated by WT and TRAC KO were ~0.3% and 0.04% overall respectively. Zero on X-axis indicates the DSB site. There was no appreciable difference between the number of WT and TRAC indels using either Cas12a or Cas9 enzymes. **D** shows off-targeting potential of CRAFT crRNA sequence revealing no exonic off-target matches (mismatch n = 4). **E** Flow cytometric data shows TCR and CD3 protein expression on primary T-cell surface after TRAC gene only KO, TRBC gene only KO (via CRAFT crRNA), and TRAC + TRBC multiplexed gene KO (via CRAFT crRNA).

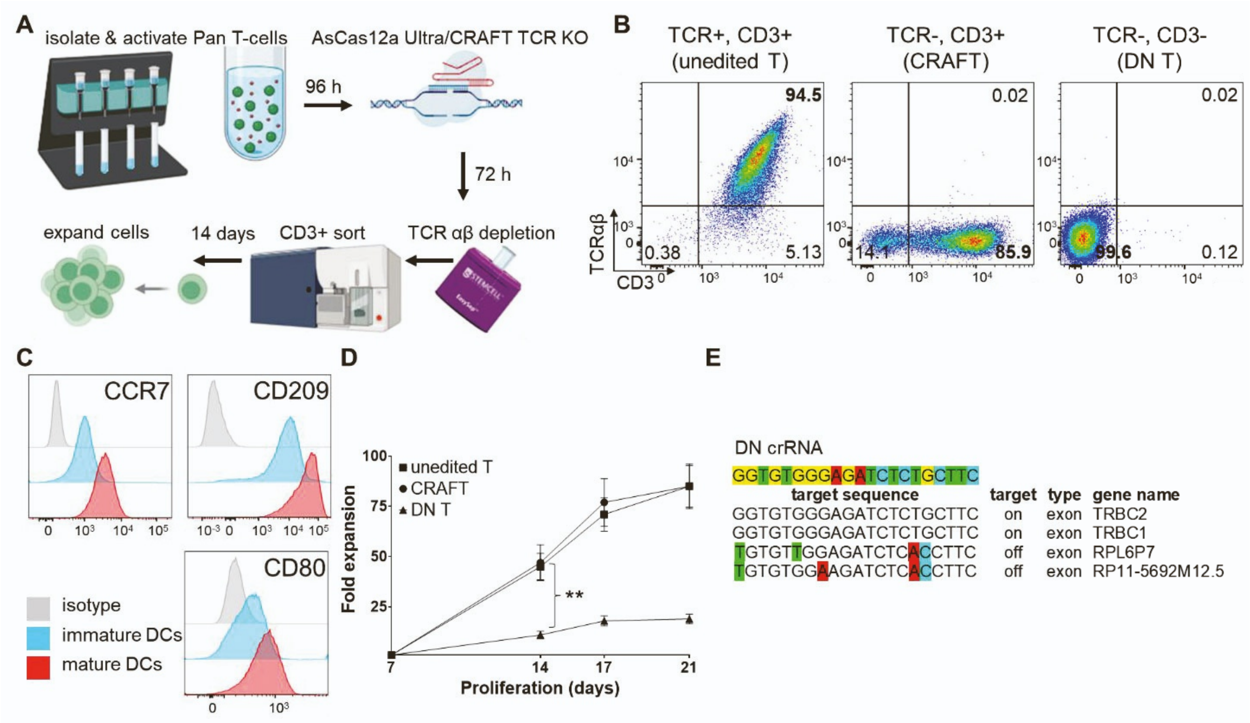

Figure S2. Generation of CRAFT-cells and comparative ex vivo fold expansion to unedited T-cells, *related to Figure 2*. **A** Schema of the timeline to generate, enrich, and expand CRAFT-cells derived from a healthy donor. The illustration was created with BioRender.com. **B** Representative flow cytometry dot plots of unedited T-cells, CRAFT-cells and TCR and CD3 double negative (DN) T-cells after expansion. **C** Flow cytometry panels showing DCs maturation markers (CCR7, CD209 and CD80) on day 9. Immature DCs did not receive polarizing agents (TNF-α, IL-1β, IL-6, and PGE2). Relevant isotype antibodies were used as controls. **D** The fold-expansion rate of unedited T-cells, CRAFT-cells, and DN T-cells up to 21 days. Mean ± SEM are plotted; multiple t-test indicating statistics on day 14 **, $P<0.01$. ($n = 6$). **E** shows off-targeting potential of TRBC (DN) crRNA sequence revealing two exonic off-target matches and the genes that would be disrupted. This guide RNA sequence was used to generate DN T-cells. The figure excludes multiple non-coding off-target candidates for the sake of conciseness and clarity.

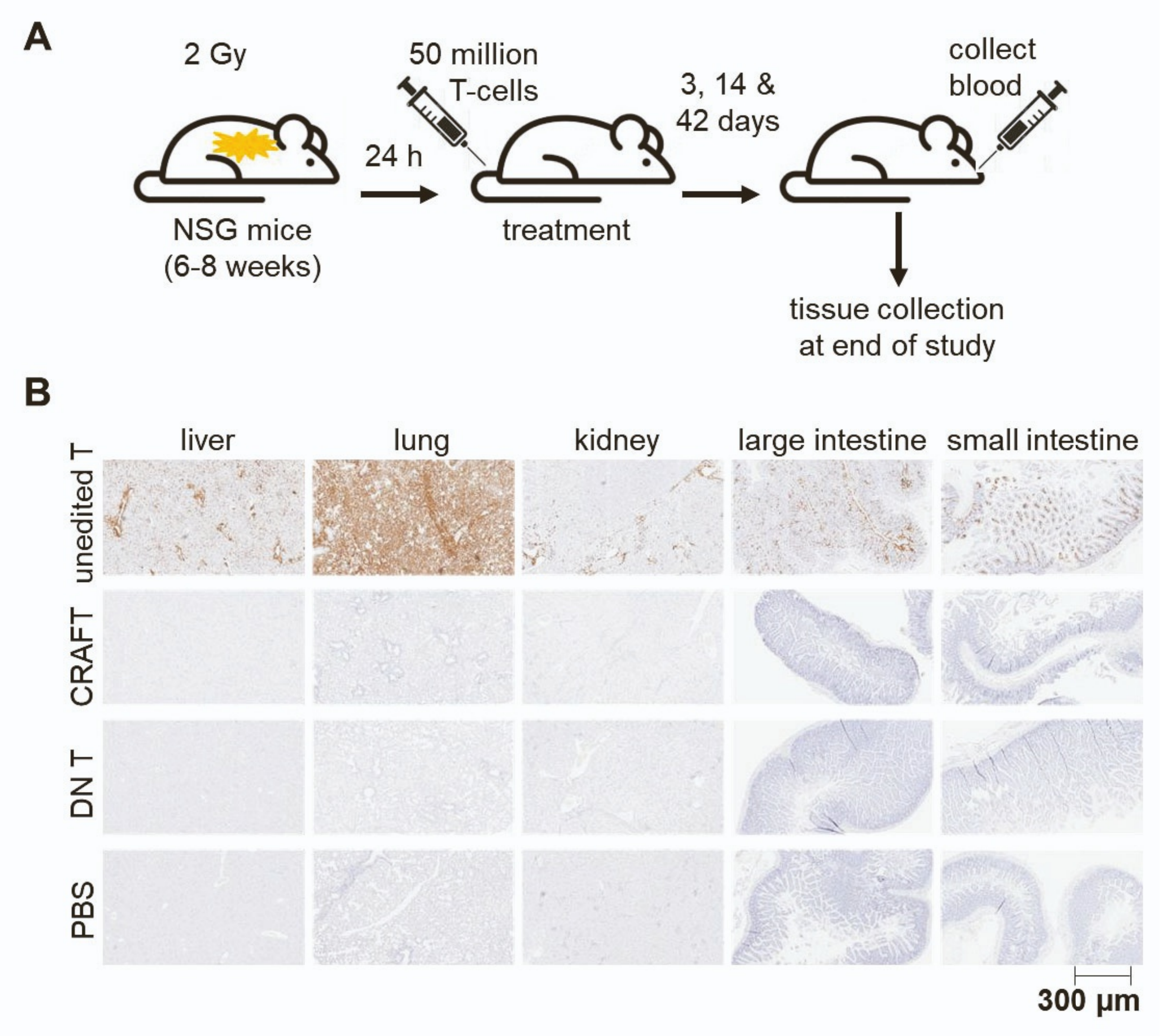

Figure S3. CRAFT-cells do not cause GVHD in immunocompromised mice, *related to Figure 3*. A Illustration of the experimental design to evaluate GVHD in NSG mice. NSG mice first received total body irradiation with a sublethal X-Ray dose of 2 Gy. 24 hours later irradiated mice were then engrafted with 50 million T-cells (treatment) of different phenotypes. Blood collections were performed on day 3, 14 and 42 after cellular treatment. Tissue for IHC was collected either at the end of study (for healthy mice) or when humane end point was reached (for moribund mice). **B** Representative immunohistochemical raw data show CD3+ cells per mouse, per study cohort (all tissues were from the same mouse per cohort). IHC was performed on all the animals that were included in various study replicates. The raw unmodified images were from the same experimental replicate. L-R: Liver, lung, kidney, large intestine, small intestine all images at 300 µm scale 10x magnification.

A

sample name	cell no. (10^6) mean ±SD	viability (%) mean ±SD	EGFR+ (%) mean ±SD	TCR- (%) mean ±SD
**unedited CAR T**	55.5 ±15	88.7 ±3	68.8 ±6	2.78 ±0.4
**CRAFT CAR T**	47.8 ±8	89.2 ±4	91.0 ±3	99.7 ±0.3
**DN CAR T**	58.5 ±21	89.7 ±5	70.6 ±4	99.8 ±0.3
**non-CAR T**	47.5 ±7	89.0 ±2	0.69 ±0.5	2.60 ±0.9

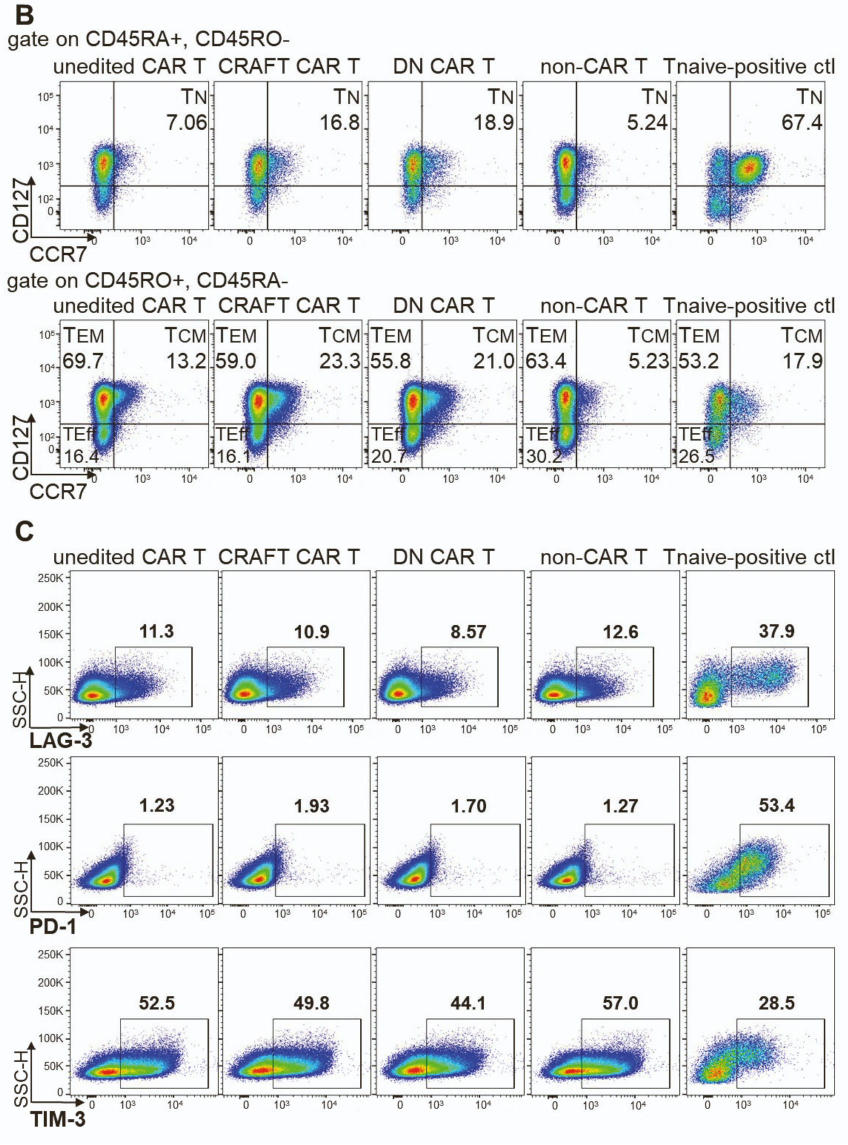

Figure S4. Comparative growth rates of all allogeneic CAR T-cell fractions, *related to Figure 4*. A Truncated EGFR incorporated in CAR construct doubles as a CAR potency marker as well as a safety switch. The mean values are reported (n = 4); error bars = ±SEM. **B** The phenotypic characterization of T-cell memory subsets was done using CD45RA and CD45RO. T-cells were further assigned memory subsets using CCR7 and CD127 within CD45RA+CD45RO– (T_N cells) and CD45RA–CD45RO+ (T_{CM}, T_{EM}, and T_{EFF} cells). The top plots depict T_N cells (unlabeled quadrants are T_{EMRA}), while the bottom plots represent T_{CM}, T_{EM}, and T_{EFF} cells. Naïve T-cells (CD14-, CD127+) were used as positive control. **C** Flow cytometric analysis comparing expression of common T-cell exhaustion markers (LAG-3, PD-1 and TIM-3) in CAR T-cell products on day 14 (final day of manufacture). T-cells re-stimulated with CD3/CD28 multiple times, were used to generate exhausted T-cell-like phenotype.

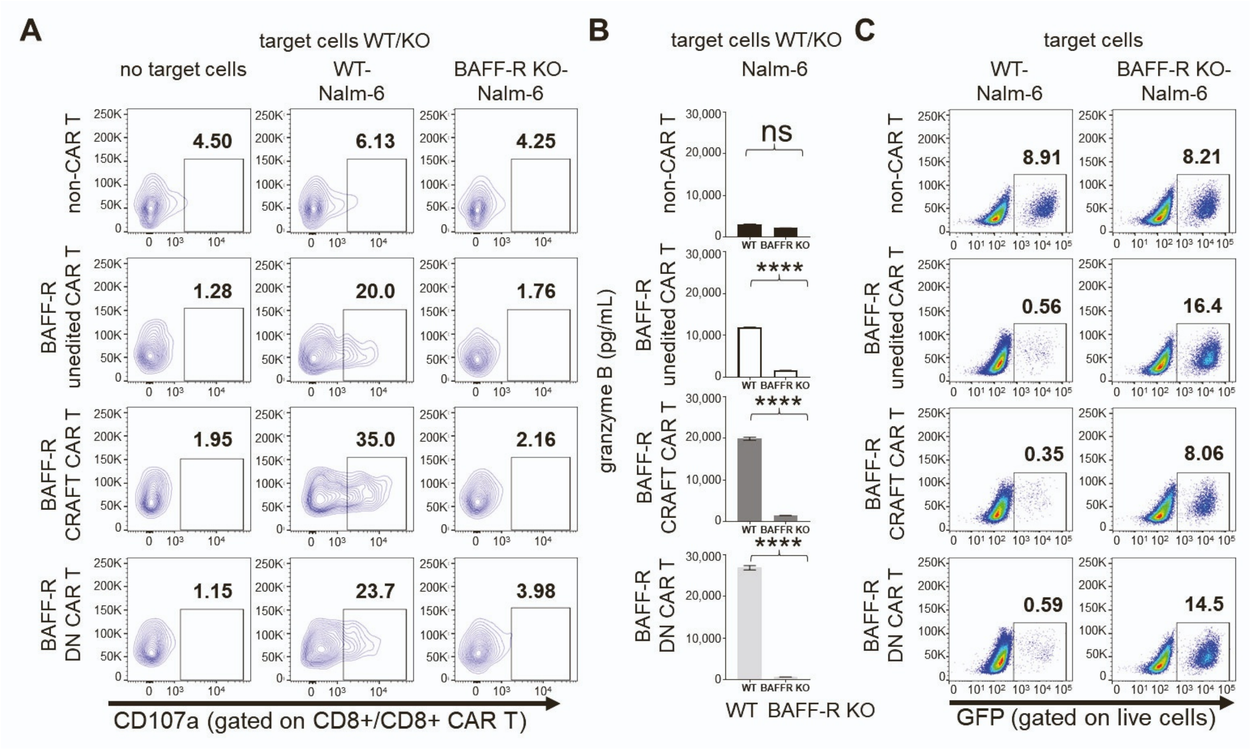

Figure S5. Novel allogeneic CD8+ BAFF-R CAR T-cells show potent, antigen-specific cytotoxicity, *related to Figures 5*. **A** Using CD107a degranulation assay, percentage of BAFF-R unedited CAR T-cells, BAFF-R CRAFT CAR T-cells, and BAFF-R DN CAR T-cells are shown to target BAFF-R Nalm-6 cells. The CAR T-cells were gated on CD8+ EGFR+, whereas non-CAR T-cells were gated on CD8+ alone (representative data included with total repeats of n = 3). **B** An ELISA showed granzyme B release (pg/mL) by the all the BAFF-R targeting CAR T-cells in response to Nalm-6 target cell lines. Non-CAR T-cells were a negative control (representative data shown for total repeats n = 3). Bar graphs plotted the means ± SEM. ****, P< 0.0001; ns, not significant. **C** The direct killing assay showed percent cytotoxicity of BAFF-R targeting CAR T-cells against Nalm-6-GFP cells. The non-CAR T-cells and the BAFF-R KO Nalm-6 target cells served as negative controls (representative data).

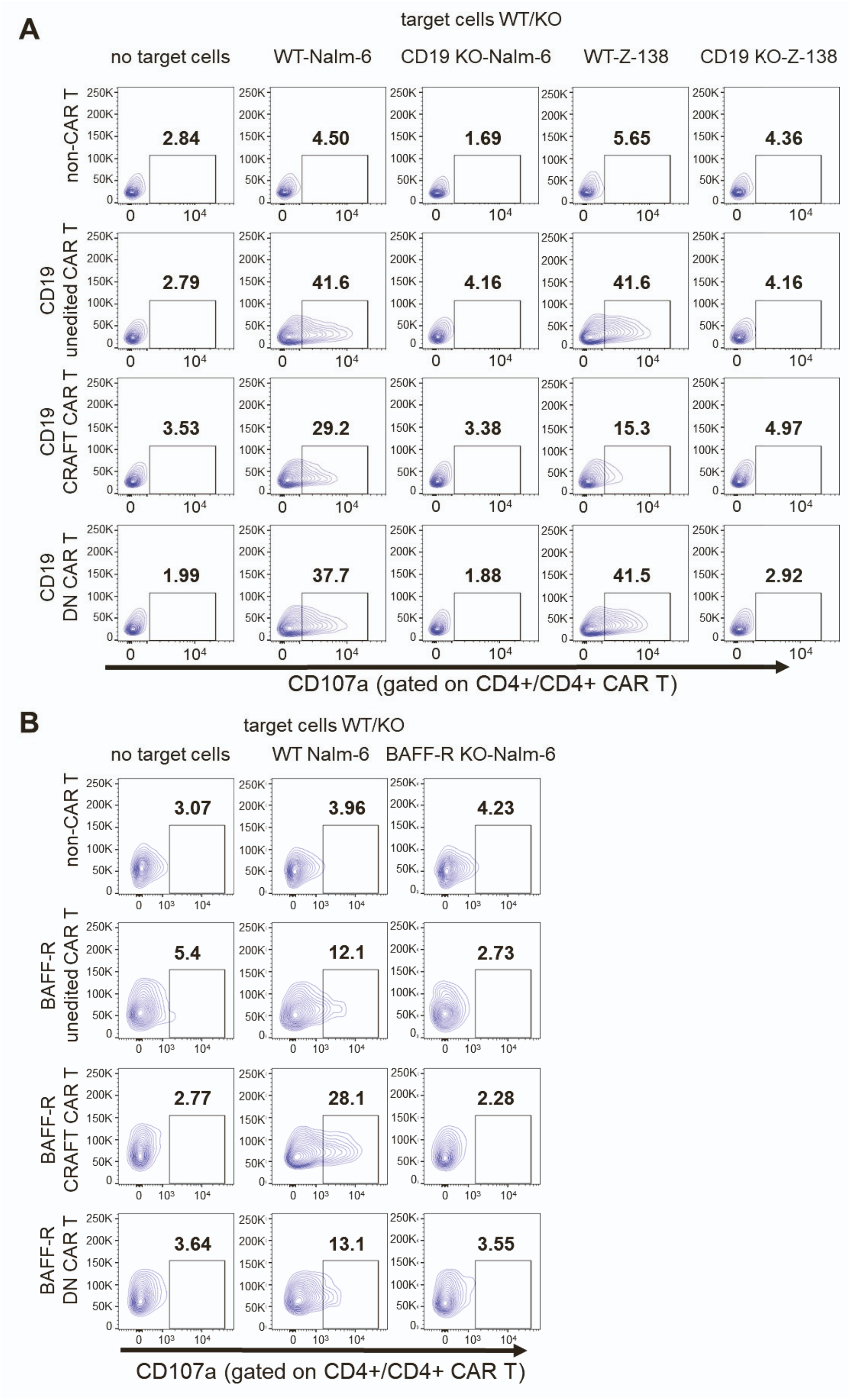

Figure S6. Novel allogeneic CD4+ CD19 CRAFT CAR and CD4+ BAFF-R CRAFT CAR show potent, antigen-specific cytotoxicity. ***Related to Figures 5.*** **A** CD107a degranulation assay shows various CD19 CAR T-cells target Nalm-6 and Z-138. The non-CAR T-cells served as a baseline/negative degranulation control. The CAR T-cells were gated on CD4+ EGFR+, whereas non-CAR T-cells were gated on CD4+ alone (representative figure, total repeats n = 3). **B** Similarly, CD107a degranulation assay using various BAFF-R CAR T-cells show targeting of Nalm-6 cells. (representative figure, total repeats n = 3).

Table S1. List of key material resources.

REAGENT OR RESOURCE	SOURCE	IDENTIFIER
**Antibodies**		
anti-human CD3ε clone UCHT1 BV605	Biolegend	300459
anti-human TCR α/β clone IP26 APC/PE	Biolegend	306718/306717
Cetuximab biotin antibody	R&D Systems	FAB9577B
anti-human APC-streptavidin antibody	BD Biosciences	554067
SYTOX™ Blue Dead Cell Stain	Invitrogen	S34857
SYTOX™ Green Ready Flow™ Reagent	Invitrogen	R37168
anti-human CD14 clone M5E2 BV650	BD Biosciences	563419
anti-human HLADR clone G46-6 PE	BD Biosciences	555812
anti-human CD11c clone SHCL-3 BV421	BD Biosciences	744434
anti-human CD209 clone DCN46 APC	BD Biosciences	551545
anti-human CCR7 clone 2-L1-A BV786	BD Biosciences	566758
anti-human CD80 clone B7-1 PerCP-Cy5.5A	BD Biosciences	567437
anti-human CD3 clone HIT3α BV605	BD Biosciences	564712
anti-human CD4 clone SK3 PE-Cy7	BD Biosciences	557852
anti-human CD8 clone SK1 APC-Cy7	BD Biosciences	348793
anti-human EGFR AY13 clone BV421/APC	Biolegend	352911/ 352906
anti-human CD107a clone H4A3 APC	BD Biosciences	560664
anti-human CD45 clone HI30 PE/BUV395	BD Biosciences	555483/563792
FxCycle™ Violet Stain (Fx Vio)	Invitrogen	F10347
Propidium Iodide Ready Flow™ Reagent	Invitrogen	R37169
**Cell lines and primary cells**		
Nalm-6 luc-GFP (WT, KO CD19, KO BAFF-R)	[25]	-
Z-138 luc-GFP (WT, KO CD19)	[25]	-
PBMC cells from healthy donor	from clinic	-
Packaged Lentivirus (>4X 10^7 MOI= 3)	in house	-
**Critical commercial assays/ kits**		
human pan T-cell isolation kit	Miltenyi Biotec	130-096-535
EasySep™ human TCR a/b depletion kit	Stemcell	17847
Human Granzyme B kit	Meso Scale Discovery	K151APDK
QIAamp DNA mini kit	Qiagen	51304
Click-iT™ EdU Cell Proliferation Kit	Thermo Fisher Scientific	C10337

**Recombinant proteins and chemicals**		
Recombinant human Interleukin-2/ Proleukin (aldesleukin)	Clinigen	NDC-76310-022-01
Recombinant human Interleukin-15	Celgeneix GMBH	1013-050
Recombinant human Interleukin-4	PeproTech	200-04
Recombinant human GM-CSF	PeproTech	300-03
Recombinant human TNFα	PeproTech	300-01A
Recombinant human Interleukin-1β	PeproTech	200-01B
Recombinant human Interleukin-6	PeproTech	200-06
PGE2	Stemcell	72192
Blinatumomab (Blincyto)	from Clinic	-
X-VIVO™ 15 media	Lonza	04-418Q
Head-inactivated human male AB plasma	Sigma-Aldrich	H3667
Dynabeads Human T-Activator CD3/CD28	Gibco	11131D
CRISPR crRNAs and sgRNA	IDT	made-to-order
Alt-R™ A.s.Cas12a/Cpf1 Ultra nuclease	IDT	10001273
Alt-R® Cpf1 Electroporation Enhancer	IDT	1076301
Alt-R™ S.p. HiFi Cas9 Nuclease V3	IDT	1081060
Alt-R® Cas9 Electroporation Enhancer	IDT	1075915
Nuclease-free 1X IDTE solution	IDT	11-05-01-13
P3 primary cell 4D 96-well nucleofector kit S	Lonza	PBP3-00675
GolgiStop™ Protein Transport Inhibitor	BD Biosciences	554724
Diluent 3	Meso Scale Discovery	R50AP
Diluent 58	Meso Scale Discovery	R50CA
Wash buffer 20X	Meso Scale Discovery	R61AA
MSD gold Read buffer B	Meso Scale Discovery	R60AM
RPMI-1640® medium	ATCC	30-2001
Iscove's Modified Dulbecco's Medium (IMDM)	ATCC	30-2005
Fetal bovine serum (FBS)	Thermo Fisher Scientific	16140071
PBS-1X	Fisher	MT21040CV
Ficol-Paque™	Cytiva	45-001-751
ACK lysis buffer	Gibco	A1049201
MACS® LS columns	Miltenyi Biotec	130-042-401
Heparin tubes	Sarstedt	20.1345.100
